# TRAIL agonists rescue mice from radiation-induced lung, skin, or esophageal injury

**DOI:** 10.1172/JCI173649

**Published:** 2025-01-14

**Authors:** Jillian Strandberg, Anna Louie, Seulki Lee, Marina Hahn, Praveen Srinivasan, Andrew George, Arielle De La Cruz, Leiqing Zhang, Liz Hernandez Borrero, Kelsey E. Huntington, Payton De La Cruz, Attila A. Seyhan, Paul P. Koffer, David E. Wazer, Thomas A. DiPetrillo, Stephanie L. Graff, Christopher G. Azzoli, Sharon I. Rounds, Andres J. Klein-Szanto, Fabio Tavora, Evgeny Yakirevich, Abbas E. Abbas, Lanlan Zhou, Wafik S. El-Deiry

**Affiliations:** 1 Laboratory of Translational Oncology and Translational Cancer Therapeutics, Warren Alpert Medical School of Brown University, Providence, Rhode Island, USA.; 2 Biomedical Engineering Graduate Group, Brown University, Providence, Rhode Island, USA.; 3 The Joint Program in Cancer Biology, Brown University and the Lifespan Health System, Providence, Rhode Island, USA.; 4 Legorreta Cancer Center, Brown University, Providence, Rhode Island, USA.; 5 Department of Surgery, Warren Alpert Medical School of Brown University and Lifespan Health System, Providence, Rhode Island, USA.; 6 D&D Pharmatech, Seongnam-si, South Korea.; 7 Department of Pathology and Laboratory Medicine, Brown University, Providence, Rhode Island, USA.; 8 Pathobiology Graduate Group, Brown University, Providence, Rhode Island, USA.; 9 Department of Radiation Oncology, Warren Alpert Medical School, Brown University and the Lifespan Health System, Providence, Rhode Island, USA.; 10 Hematology/Oncology Division, Department of Medicine, Brown University and the Lifespan Health System, Providence, Rhode Island, USA.; 11 Division of Pulmonary Medicine, Warren Alpert Medical School of Brown University and Lifespan Health System, Providence, Rhode Island, USA.; 12 Providence Veterans Administration Medical Center, Providence, Rhode Island, USA.; 13 Fox Chase Cancer Center, Philadelphia, Pennsylvania, USA.; 14 Argos Laboratory, Universidade Federal do Ceará Fortaleza, Ceará, Brazil.; 15 Division of Thoracic Surgery, Department of Surgery, Warren Alpert Medical School of Brown University and Lifespan Health System, Providence, Rhode Island, USA.

**Keywords:** Oncology, Cancer, Innate immunity, Radiation therapy

## Abstract

Radiotherapy can be limited by pneumonitis, which is impacted by innate immunity, including pathways regulated by TRAIL death receptor DR5. We investigated whether DR5 agonists could rescue mice from toxic effects of radiation and found that 2 different agonists, parenteral PEGylated trimeric TRAIL (TLY012) and oral TRAIL-inducing compound (TIC10/ONC201), could reduce pneumonitis, alveolar wall thickness, and oxygen desaturation. Lung protection extended to late effects of radiation including less fibrosis at 22 weeks in TLY012-rescued survivors versus unrescued surviving irradiated mice. Wild-type orthotopic breast tumor–bearing mice receiving 20 Gy thoracic radiation were protected from pneumonitis with disappearance of tumors. At the molecular level, radioprotection appeared to be due to inhibition of CCL22, a macrophage-derived chemokine previously associated with radiation pneumonitis and pulmonary fibrosis. Treatment with anti-CCL22 reduced lung injury in vivo but less so than TLY012. Pneumonitis severity was worse in female versus male mice, and this was associated with increased expression of X-linked TLR7. Irradiated mice had reduced esophagitis characterized by reduced epithelial disruption and muscularis externa thickness following treatment with the ONC201 analog ONC212. The discovery that short-term treatment with TRAIL pathway agonists effectively rescues animals from pneumonitis, dermatitis, and esophagitis following high doses of thoracic radiation exposure has important translational implications.

## Abstract 2

Prevention of lethality, severe pneumonitis, lung fibrosis, skin dermatitis and esophagitis post-g-irradiation by short-term treatment with innate immune TRAIL pathway agonists

## Introduction

Cancer therapy is limited by toxicities that impact quality of life and cause morbidity. A general goal in medicine has been to develop better-tolerated therapeutics. Radiation therapy has efficacy in local control of cancer and patient survival outcomes in multiple tumor types (1, 2). However, toxicity toward normal tissues has been a challenge in patient treatment (3). Other cancer therapeutics including bleomycin and immune checkpoint blockade cause morbidity, can interfere with continuing treatment as a result of lung inflammation and injury, and ultimately contribute to mortality (4, 5).

The late effects of radiation involve inflammation, fibrosis, and morbidity, thus limiting use of further treatments in patients with symptoms (6, 7). Radiation-induced pneumonitis develops in approximately 10%–30% of thoracic cancer patients (8). While high-dose radiotherapy can benefit patients with lung cancer, the severe toxicity and dose fractionation are inconvenient (9–11). Radiotherapy is often combined with immunotherapy to treat cancer, and this greatly increases risk of serious lung injury (12, 13). Strategies are needed to reduce serious toxicities that limit cancer treatment to improve patient care. There is a need to develop radiation countermeasures for use in the unfortunate event of a “dirty bomb,” nuclear power plant leak, or other unanticipated exposure to high radiation doses (14, 15). Such strategies could be used to achieve radioprotection if agents are effective after radiation exposure.

Esophageal cancer is common worldwide with more than 600,000 new cases each year and 540,000 deaths (16). While squamous cell cancer is the most common esophageal cancer worldwide, in the United States adenocarcinoma accounts for most cases (17, 18). Multimodal chemoradiotherapy is used for advanced disease and preoperatively for locally advanced disease (19–22). Definitive radiotherapy improves outcomes in both settings (23), and radiotherapy alone has been suggested for some older individuals (24). Chemoradiotherapy or radiotherapy is combined with immunotherapy to improve outcomes in esophageal cancer (25–27). Radiotherapy with curative intent or palliation in esophageal cancer or head and neck cancer is often associated with inflammation, debilitating esophageal injury, pain, difficulty swallowing, and dehydration (28–33).

We discovered TRAIL death receptor DR5 as a direct transcriptional target of the p53 tumor suppressor protein (34, 35). p53 protein is central to the cellular DNA damage response inflicted by radiation damage. Stabilization of p53 protein after DNA damage activates genes encoding proteins such as p21(WAF1) that mediate cell cycle arrest and repair of DNA damage (36, 37). Repair of damage is essential for cell survival without cancer. DR5 is involved in cell death after chemotherapy or radiotherapy of tumors. The TRAIL pathway through DR5 is part of the host innate immune system that suppresses cancer and metastases (38).

We demonstrated that *Dr5* (also known as *Trail-R2* or *Tnfrsf10b*) gene deletion in mice reduces cell death in multiple organs after lethal γ-irradiation (39), and that a sublethal dose of whole-body γ-irradiation causes inflammatory lesions and fibrosis in multiple organs, including in the lungs of irradiated mice (40). This results in lethality of irradiated *Dr5^–/–^* mice 6–8 months after irradiation. Histological analysis of lung tissues from *Dr5^–/–^* mice suggested similarity to late effects of radiation in patients who receive thoracic radiation and develop pneumonitis with deposition of collagen and fibronectin (40).

We have known for more than 15 years that a primary strategy for rescue from the toxic effects of lung radiation might involve using TRAIL pathway agonists. With the availability of new approaches to stimulate the TRAIL innate immune pathway (41–44), we attempted to rescue mice from radiation toxicities. The multiple available TRAIL innate immune pathway agonists include TRAIL-inducing compound #10 (TIC10/ONC201), currently in clinical trials for various tumor types (41, 44, 45), and TLY012, a novel PEGylated trimeric TRAIL with antifibrotic properties (43). ONC212, a potent ONC201 analog, has preclinical efficacy in pancreatic cancer, liver cancer, and melanoma (46–48). DR5 agonist antibodies are being developed for cancer by pharmaceutical companies. We show that short-term TRAIL pathway agonist treatment for 2 weeks at or after radiation exposure rescues mice from pneumonitis or dermatitis following 20 Gy thoracic radiation exposure. We also observed reduced severity of radiation-induced esophagitis following treatment with ONC212 after a 20 Gy thoracic x-ray irradiation dose in mice. ONC212 rescue was associated with serum downregulation of epidermal growth factor (EGF), IL-16, chemokine ligand 3 (CCL3), CCL7, and prolactin, suggesting decreased inflammation, while upregulation of CCL5, CXCL12, CCL22, and insulin-like growth factor 1 (IGF-1) is consistent with healing from radiation damage. Our findings merit further investigation and clinical translation to reduce morbidity from radiation injury.

## Results

### TRAIL pathway agonists protect mice from lung injury and fibrosis following high-dose thoracic ionizing radiation.

**Figure 1:**
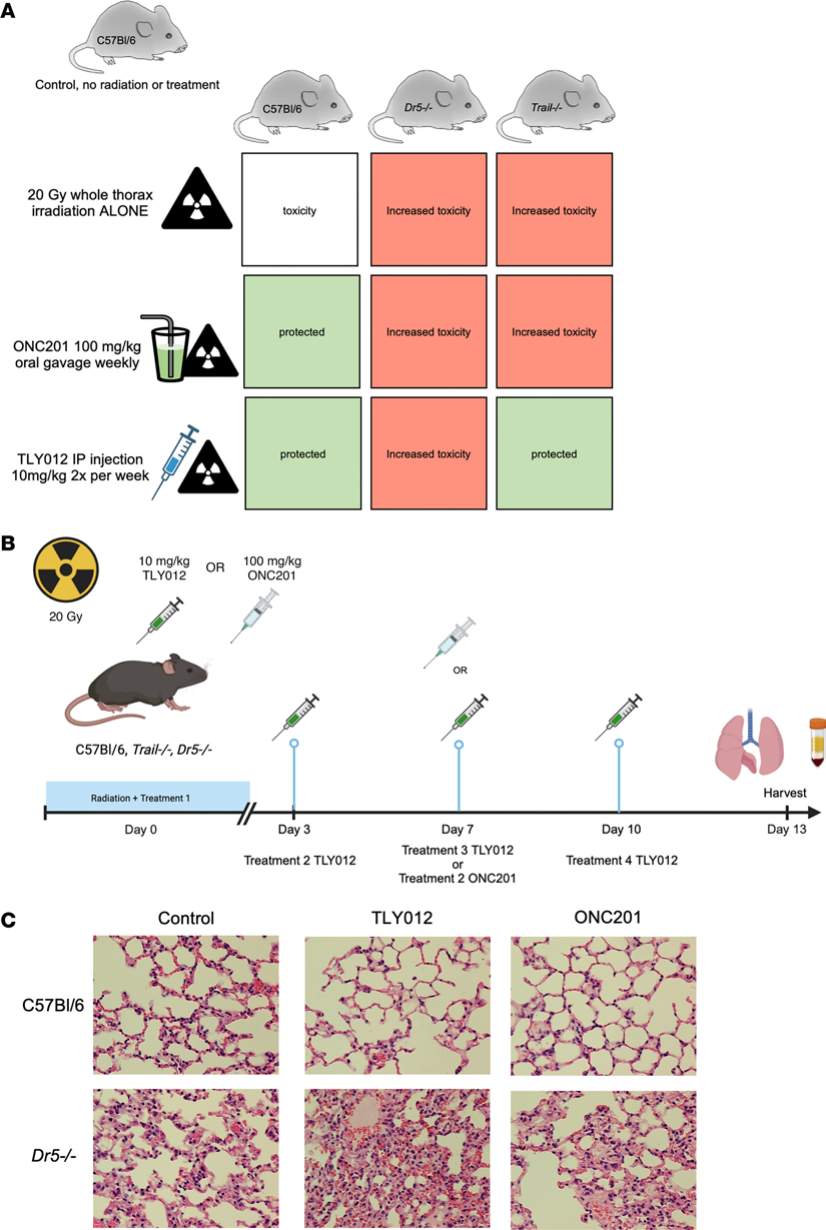
Experimental design and preliminary results of the mouse strains and treatment cohorts. (**A**) Hypothesized outcomes regarding lung protection of C57BL/6, *Dr5^–/–^*, and *Trail^–/–^* mice given a whole-thorax x-ray irradiation dose of 20 Gy and either 100 mg/kg of ONC201 weekly, 10 mg/kg of TLY012 twice weekly, or no treatment. (**B**) Experimental timeline. Mice received their first treatment an hour before radiation, and were treated with either ONC201 once a week or TLY012 twice weekly until sacrifice on day 13 after irradiation. (Created in BioRender. Strandberg J. 2024. https://BioRender.com/p52s542.) (**C**) First observations of suppression of radiation pneumonitis in H&E-stained lung tissue of male mice by short-term treatment with TRAIL pathway agonists as indicated (*n* = 2 per treatment per genotype) (original magnification, ×20).

We investigated toxicities of high-dose thoracic radiation in wild-type (WT), *Dr5^–/–^*, or *Trail^–/–^* C57BL/6 mice to test outcomes predicted in Figure 1A. We hypothesized that *Dr5^–/–^* mice would have severe radiation-induced pneumonitis and would not be rescued by either TRAIL or TIC10/ONC201 (40). We also hypothesized that TLY012 would rescue pneumonitis in *Trail^–/–^* mice whereas TIC10/ONC201 would not because the *Trail* gene upregulated by the drug is deleted.

A preliminary, short-term treatment feasibility experiment was conducted to ensure some mouse survival after 20 Gy of thoracic irradiation (Figure 1B). The experiment was performed with WT and *Dr5^–/–^* mice irradiated with 20 Gy, followed by weekly doses of TIC10/ONC201 (100 mg/kg) or twice-weekly doses of TLY012 (10 mg/kg) for 2 weeks. Unexpectedly, given the brevity of attempted rescue treatment, as shown in Figure 1C, we observed qualitative evidence of protection from pneumonitis in C57BL/6 mice treated with either TLY012 or ONC201 versus control. Lung inflammation in irradiated mice was substantially worse in *Dr5^–/–^* mice regardless of treatment with TLY012 or ONC201 (Figure 1C). It was surprising that few doses of either TRAIL pathway agonist effectively protected against severe acute radiation-induced lung injury. Lack of protection of *Dr5^–/–^* mice and the appearance of a more severe phenotype are consistent with our previous observations (40). No obvious toxicities were observed in duodenum, liver, or heart (Supplemental Figure 1, A–C; supplemental material available online with this article; https://doi.org/10.1172/JCI173649DS1).

There were no differences in cellularity in bone marrow between TLY012- or ONC201-treated and untreated groups (Supplemental Figure 1D). Complete blood counts taken in female C57BL/6 mice at 1 week and 2 weeks after irradiation (Supplemental Table 1) and from male and female *Trail^–/–^* mice 2 weeks after irradiation (Supplemental Table 2) showed no statistical differences across groups treated with TLY012 or control. Toxicity was also monitored by weighing of the mice twice weekly (Supplemental Figure 1, E–G). In all experiments, lungs were not reinflated postmortem, as it was determined there were no substantial visual differences between inflated and uninflated lungs (Supplemental Figure 1H).

### Rescue of WT or Trail^–/–^ mice but not Dr5^–/–^ mice from radiation pneumonitis by TLY012.

**Figure 2:**
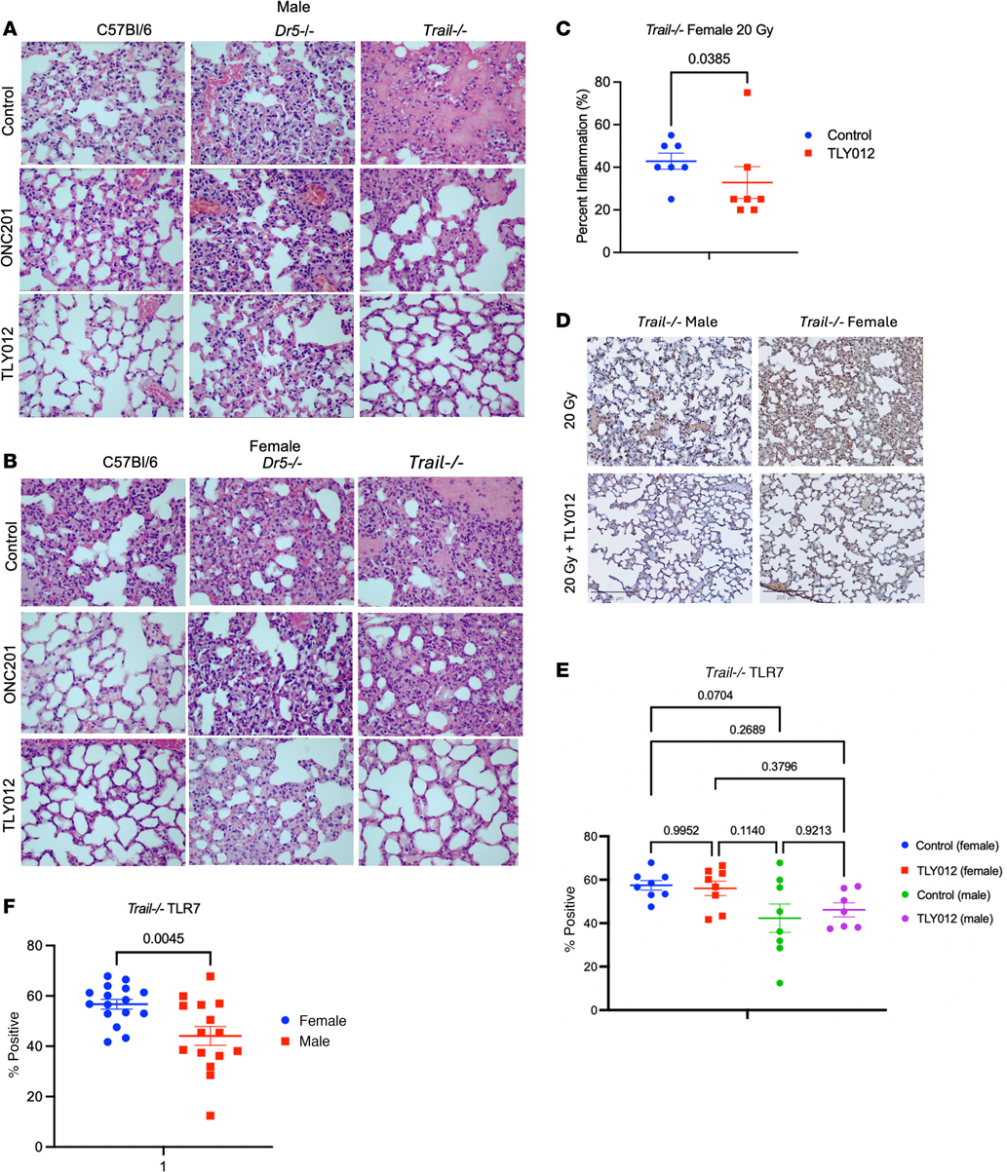
Protection of mice from radiation pneumonitis and its dependence on genetic strain and TRAIL pathway agonist. (**A** and **B**) Representative H&E stains of lung tissue from male and female mice from C57BL/6, *Dr5^–/–^*, and *Trail^–/–^* backgrounds treated with ONC201, TLY012, or control gavage (*n* = 2 per sex per genotype per treatment) 13 days after irradiation (original magnification, ×40). (**C**) Quantification of percentage inflammation of *Trail^–/–^* female mice treated with TLY012 or control (*n* = 7 per treatment per group) showed significant decrease (*P* = 0.0385) in inflammation 2 weeks after thoracic irradiation of 20 Gy (1-tailed Mann-Whitney test). (**D**) Representative images of IHC staining of TLR7 in lung tissue from *Trail^–/–^* female and male mice at 2 weeks after irradiation (original magnification, ×10). Scale bars: 200 μm. (**E**) Quantification of positive staining of TLR7 in mouse lung separated by treatment group (*n* = 8 per treatment per group) (1-way ANOVA with Tukey’s post hoc test). (**F**) When separated by sex (*n* = 16 per sex), there was a statistically significant increase in female mice (*P* = 0.0045). Values are mean ± SEM (unpaired 2-tailed *t* test).

We tested the prediction that *Trail^–/–^* mice would be rescued by TRAIL (TLY012) but not by TIC10/ONC201 (Figure 1A). As predicted, short-term TLY012 treatment over 2 weeks rescued 20-Gy-irradiated male (Figure 2A) and female (Figure 2B) mice, while ONC201 did not. This is consistent with the notion that if the *TRAIL* gene is not present, then ONC201 would not be expected to increase its expression for pneumonitis rescue. The results in Figure 2, A and B, demonstrate not only severe lung inflammation in 20-Gy-irradiated *Trail^–/–^* mice, but also their rescue by TLY012. As hypothesized, we observed no rescue of *Dr5^–/–^* 20-Gy-irradiated lungs by either TLY012 or ONC201 (Figure 2, A and B).

Percentage inflammation, quantified by a blinded animal pathologist, determined there was increased toxicity in female *Dr5^–/–^* mice (Supplemental Figure 2, A and B), which was further exacerbated in female *Trail^–/–^* mice (Figure 2C). A statistically significant decrease in inflammation was observed in *Trail^–/–^* female mice treated with TLY012 twice weekly for 2 weeks after thoracic irradiation of 20 Gy (*P* = 0.0385). While not to a statistically significant degree (*P* = 0.0924), percentage fibrosis decreased in TLY012-treated female C57BL/6 mice (Supplemental Figure 2A). A small decrease in interstitial cell count was observed in female WT but not *Dr5^–/–^* mice (Supplemental Figure 2, C and D).

Unexpectedly, the severity of radiation-induced pneumonitis was worse in female versus male mice. Prior studies have noted more toxicity in either male rats or patients (49–51). In patients, it may be that more men had lung cancer and were treated with radiation.

### Association between TLR7 and sex differences in development and treatment of radiation pneumonitis.

Upon histological examination, female mice had a more pronounced inflammatory response to radiation, particularly *Trail^–/–^* females (Figure 2B). Female mice experienced greater weight loss after radiation compared with male mice (Supplemental Figure 1, D–F). Immunohistochemical staining for Toll-like receptor 7 (TLR7) in *Trail^–/–^* mice (Figure 2D) showed that while there was no statistically significant difference between treatment groups (Figure 2E), when treatment groups were combined within male and female cohorts, a significant increase in TLR7 expression was seen in the female cohort (*P* = 0.0045) (Figure 2F). This implies that a gene dosage effect may be at play and points to an association between TLR7 and sex differences in radiation pneumonitis.

### Decreased collagen production in mice after 18 Gy thoracic radiation and TLY012.

**Figure 3:**
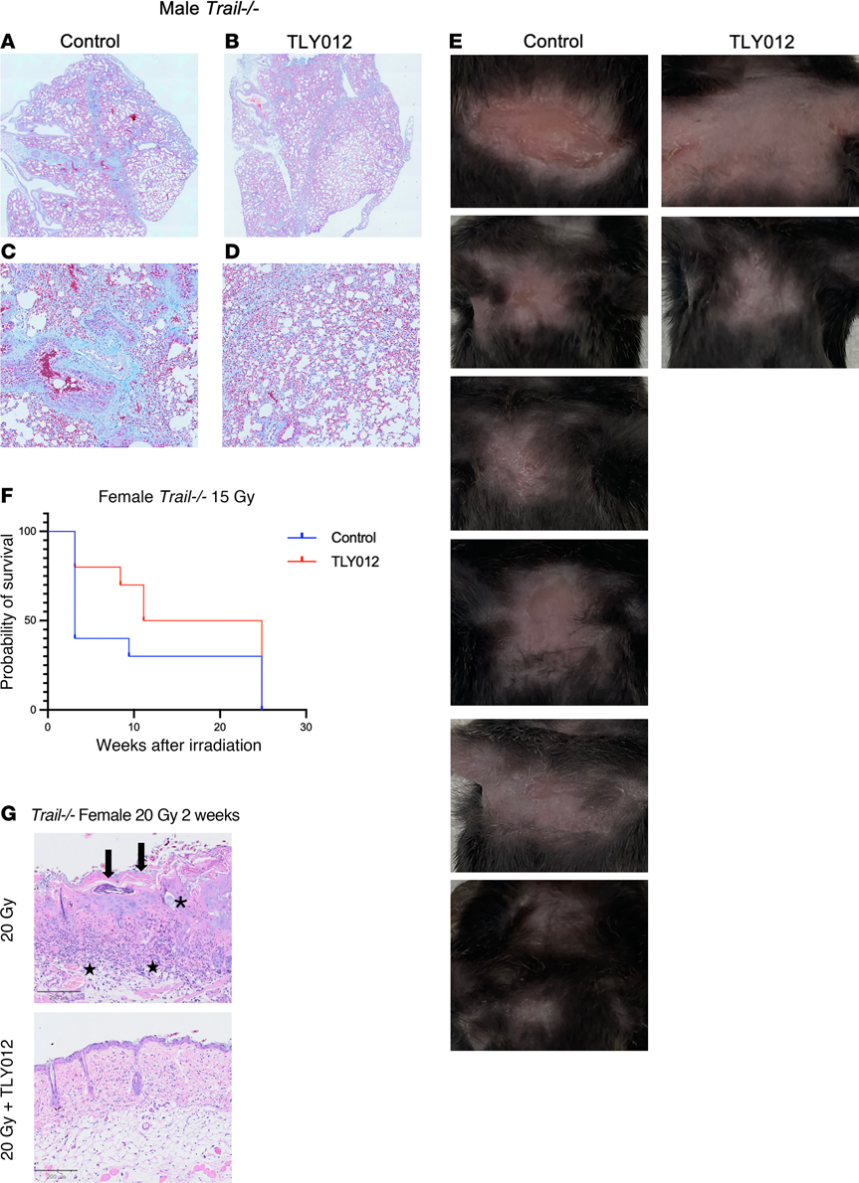
Rescue from long-term effects of radiation pneumonitis and radiation dermatitis by treatment of TLY012 up to 22 weeks after irradiation. (**A**–**D**) Male *Trail^–/–^* mice were treated with a single thoracic x-ray irradiation dose of 18 Gy and treated with TLY012 (**B** and **D**) or controls (**A** and **C**) for 22 weeks after radiation (*n* = 3, *n* = 2). Lung tissue stained with Masson’s trichrome and imaged at ×10 (**A** and **B**) or ×20 (**C** and **D**) original magnification. Red, muscle fibers; bright blue, collagen; dark red/blue, nuclei. (**E**) Mice that developed radiation burns 3 weeks after irradiation on the chest were euthanized (control *n* = 6, TLY012 *n* = 2). (**F**) Kaplan-Meier curve of *Trail^–/–^* female mice rescued from radiation dermatitis after a single whole-thorax x-ray irradiation dose of 15 Gy treated with TLY012 compared with control (*n* = 10 per treatment per group). (**G**) Representative H&E images (original magnification, ×10) of skin from chest of female *Trail^–/–^* mice 2 weeks after irradiation. Epidermal necrosis (arrows), subepidermal cleft (asterisk), and dermal inflammation involving superficial dermis and focally subcutaneous adipose tissue (stars) were observed in the control group (original magnification, ×20). Scale bars: 200 μm.

To examine late effects of thoracic radiation, we designed an experiment in which the single radiation dose was lowered to 18 Gy in male *Trail^–/–^* mice. For 22 weeks after radiation, mice either were treated with 10 mg/kg of TLY012 twice weekly or remained control. At 22 weeks, when mice reached criteria for euthanasia, they were sacrificed, and lung tissue was harvested. Lung tissue slides were stained with Masson’s trichrome and imaged at ×10 on an Olympus VS200 slide scanner (Figure 3). Upon analysis, there was substantially more light blue staining in control irradiated mice (Figure 3, A and C) versus TLY012-treated mice (Figure 3, B and D). Decreased collagen deposition in TLY012-treated mice suggests decreased inflammation and rescue from late effects of radiation pneumonitis.

### Rescue from radiation dermatitis in TLY012-treated Trail^–/–^ mice.

Female *Trail^–/–^* mice given a single whole-thorax x-ray dose of 15 Gy (*n* = 10 per treatment per group) developed radiation dermatitis in the chest area at about 3 weeks after irradiation (Figure 3E). These burns evolved rapidly over 24–48 hours once they started to develop, and the affected mice were euthanized per IACUC protocol. This unforeseen radiation toxicity was observed in *Trail^–/–^* mice and not in WT mice at the doses used. Of the *Trail^–/–^* mice that developed radiation dermatitis, 6 were control mice and 2 were TLY012-treated mice (Figure 3, E and F). Thus, TLY012 provided a protective effect against radiation-induced skin damage, as fewer TLY012-treated mice displayed severe rapidly evolving dermatitis.

When this experiment was repeated in *Trail^–/–^* male and female mice (*n* = 8 per sex per treatment per group) with 20 Gy of thoracic irradiation, histopathological examination of skin at 2 weeks after irradiation revealed that control mice in both male and female cohorts had more inflammation, including the presence of subepidermal clefts and eosinophils (Figure 3G). One of the female mice in the control group experienced epidermal necrosis, and another mouse had neutrophils in the dermis layer (Figure 3G). In addition to protection from radiation dermatitis, up to 13 weeks after irradiation, TLY012-treated mice had greater survival compared with controls (Figure 3F).

### TLY012 protects from lethal radiation pneumonitis in Trail^–/–^ mice.

We investigated whether TLY012 could rescue mice from lethality of thoracic irradiation (Supplemental Figure 3). We observed rescue of *Trail^–/–^* male mice treated with 10 mg/kg of TLY012 after an 18 Gy chest irradiation (Supplemental Figure 3A). For female *Trail^–/–^* mice we had to reduce the radiation dose to 15 Gy to observe substantial protection from lethality (Supplemental Figure 3B). There was no protection from lethality by TLY012 of C57BL/6 female mice after 25 Gy (Supplemental Figure 3C).

### Cytokine alterations in TLY012-treated mice.

We examined patterns of cytokines in C57BL/6, *Dr5^–/–^*, and *Trail^–/–^* mice irradiated and treated with either TLY012 or ONC201 or control (Supplemental Figure 4, E and F). We observed IGF-1 upregulation by ONC201, while TLY012 increased CXCL1, IL-6, GDF-15, MMP-8, and CCL3/MIP-1α (Supplemental Figure 4, E and F). Increased FGF-basic was noted with either TLY012 or ONC201. Prior work implicated other cytokines, including TGF-β, IL-6, and TNF (52). Our results provide insights into immune factors with altered secretion patterns during rescue with pneumonitis-preventing agents that activate the TRAIL pathway.

### Reduced inflammation and DNA damage in lung-irradiated versus TLY012-rescued mice.

Examination of lung tissue in irradiated C57BL/6 mice revealed that while TLY012 or ONC201 could rescue mice from radiation-induced pneumonitis (Figure 2), there was no difference in T cell infiltration as judged by the pan–T cell marker CD3ε (Supplemental Figure 4A) or the DNA double-strand break marker γ-H2AX (Supplemental Figure 4B) within lung areas deemed as normal tissue or inflamed with pneumonitis. Overall, there were fewer areas of pneumonitis in rescued mice (Figure 2) but no difference in marker expression in analyzed healthy tissue or inflamed tissue in control versus treated mice (Supplemental Figure 4, A and B). Similarly, there were no differences in NF-κB or cell proliferation (Ki67) between irradiated controls and irradiated rescued lung tissues (Supplemental Figure 4, C and D), although there were greater areas of acute inflammation in irradiated versus TRAIL pathway agonist–rescued mice. There was less collagen deposition in rescued lungs (Supplemental Figure 4E). Overall, there was less inflammation, DNA damage, and collagen deposition in rescued lungs, although in any residual inflamed tissues there were no differences between irradiated and TRAIL pathway agonist–rescued mice. While p53 expression appears similar across treatment groups, there was an observed increase in p53 expression in female mice of all 3 genotypes following irradiation (Supplemental Figure 4, F–H). Cleaved caspase-3/8 staining in *Trail^–/–^* male and female mice at 2 weeks after irradiation showed evidence of apoptosis in both TLY012-treated and untreated mouse lungs (Supplemental Figure 4, I–L).

### Altered mRNA levels of genes related to inflammation and immune response.

To determine changes in immune response between TLY012-treated mice and control mice after a dose of 20 Gy thoracic irradiation, a NanoString PanCancer Immune Profiling panel was used to analyze mRNA from mouse lung tissue. Sixteen genes had statistically significant differential expression between the TLY012-treated and control radiation-only groups. *Tnfsf10*, *Klra7*, *Ccl6*, *Tmem173*, *RelB*, *HerC6*, and *IL1RL2* were among the top upregulated differentially expressed genes (DEGs) in the TLY012-treated relative to the control group, while *Dock9*, *Mapk8*, *H2-Q2*, *PTGS2*, *RAET1A*, *BCL6*, *FoxJ1*, *IKZF2*, and *RRAD* were among the top downregulated DEGs (Supplemental Figure 5, A and B). NanoString pathway changes between TLY012-treated and control mice were examined. There were significant increases in “antigen processing” (Supplemental Figure 5, D and E) and “interferon” (Supplemental Figure 5, F and G) pathways. When the TLY012-treated and control groups were further separated into male and female cohorts, there were significant increases in “antigen processing,” “MHC,” and “dendritic cell functions” pathways and decreased “basic cell functions” pathway in female TLY012-treated mice.

In addition to NanoString PanCancer Immune Profiling, serum cytokine analysis was performed. The cytokines that had the greatest differential expression in TLY012-treated mice were decreased CCL3/MIP-1α and GDF-15 and increased IL-1β (Supplemental Figure 5C).

### Orthotopic breast tumor–bearing immune-competent mice receiving 20 Gy thoracic radiation are protected from pneumonitis while showing elimination of tumors.

**Figure 4:**
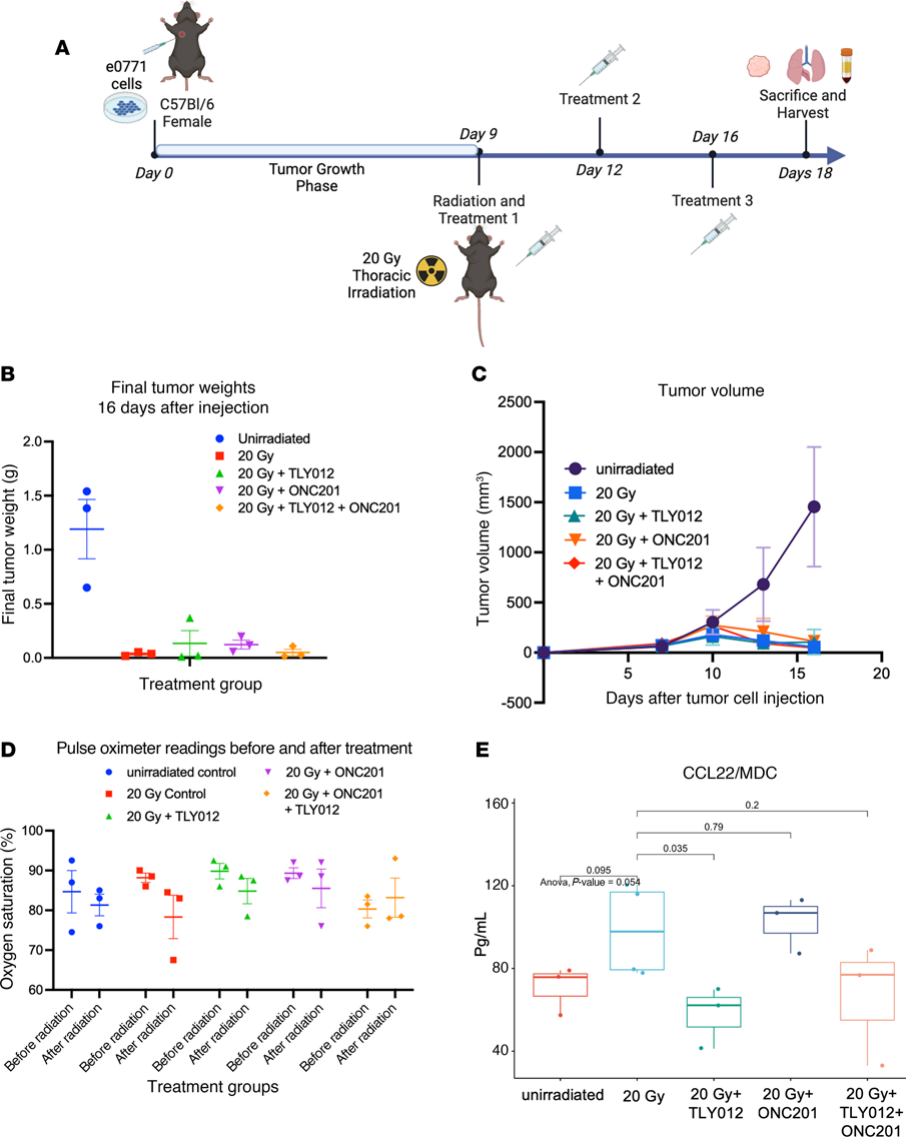
Efficacy of chest irradiation using an orthotopic immune-competent breast cancer model with prevention of pneumonitis by TLY012 and reduction of CCL22. (**A**) Experimental timeline. Female C57BL/6 mice orthotopically injected with e0771 on day 0 and when tumors reached 2–5 mm in size mice were irradiated with a whole-thorax irradiation dose of 20 Gy and treated with either TLY012, ONC201, or the combination (*n* = 3–4 per treatment per group). (Created in BioRender. Strandberg J. 2024. https://BioRender.com/c65v064.) (**B** and **C**) Tumors were removed after mice were euthanized 18 days after cell injection, and weight and volume were calculated. (**D**) Pulse oximetry readings before radiation and 9 days after radiation showed oxygen saturation more conserved in the TLY012-treated group compared with irradiated controls. Values are mean ± SEM. (**E**) Statistical analysis of cytokine fold change showed significant decrease in levels of MDC/CCL22 (*P* = 0.035) in comparison with irradiated controls (1-way ANOVA with Tukey’s post hoc test).

We conducted an experiment to determine whether TLY012, ONC201, or the combination used to prevent radiation pneumonitis might interfere with antitumor effects of radiation treatment using an orthotopically implanted breast cancer in immune-competent mice. We did not expect either ONC201, TLY012, or the combination to block antitumor efficacy given prior work (53, 54), but needed to formally show this in the radiation pneumonitis context. Murine breast cancer e0771 cells were orthotopically injected into mammary fat pad 2 of immune-competent C57BL/6 mice, which were subsequently given 20 Gy of radiation to the chest on day 9. Mice received TLY012, ONC201, the combination, or no further treatment on days 9, 12, and 16, and then mice were sacrificed on day 18 (Figure 4A) as they ultimately began to lose more than 20% body weight in most treatment groups (Supplemental Figure 6A). We observed an antitumor effect of radiation with no evidence that ONC201, TLY012, or their combination reduces treatment efficacy (Figure 4, B and C). Oxygen saturation was reduced in treated mice versus unirradiated mice, and this was partially rescued by TLY012 or ONC201 versus control irradiated mice (Figure 4D). Rescue from radiation pneumonitis was more potent by TLY012 than ONC201. The combination of TLY012 plus ONC201, while effective, did not improve protection from pneumonitis versus single treatments under the experimental conditions (Supplemental Figure 6B). Through cytokine analysis, we found differences between control and treatment groups (Supplemental Figure 6C). There was significant reduction in CCL22/MDC levels in TLY012-treated irradiated tumor-bearing mice. CCL22 (Figure 4E) is a macrophage-derived chemokine previously associated with radiation pneumonitis and pulmonary fibrosis (55).

### Anti-CCL22 provides a mild protective effect against radiation pneumonitis.

**Figure 5:**
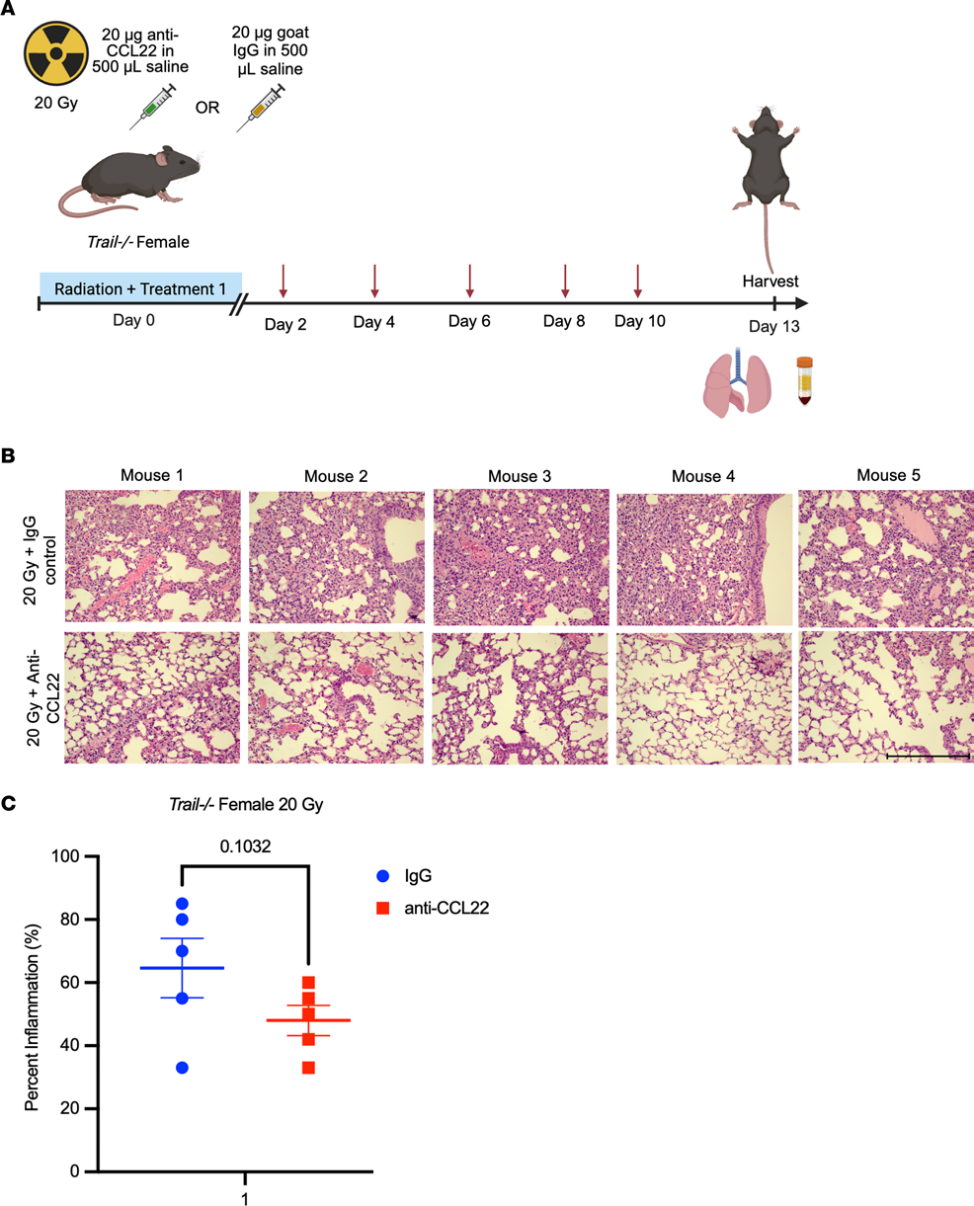
Treatment with anti-CCL22 in *Trail^–/–^* mice provided partial rescue from radiation pneumonitis. (**A**) Experimental timeline of *Trail^–/–^* female mice treated with 20 μg of anti-CCL22 in 500 μL of saline or 20 μg of goat IgG in 500 μL of saline every other day for 2 weeks (*n* = 5 per treatment per group). (Created in BioRender. Strandberg J. 2024. https://BioRender.com/j52q830.) (**B**) H&E images of each mouse 2 weeks after thoracic irradiation (original magnification, ×20). Scale bar: 100 μm. (**C**) Quantification of inflammatory scores provided by a blinded pathologist showed decrease in inflammation in *Trail^–/–^* female mice treated with anti-CCL22 (*n* = 5 per treatment per group) but not to a significant extent (1-tailed Mann-Whitney test) (*P* = 0.1032). Values are mean ± SEM.

To further investigate the effects of CCL22 suppression on rescue from radiation pneumonitis, we conducted an experiment in which female *Trail^–/–^* mice received either anti-CCL22 or control IgG treatment every other day for 2 weeks after a single whole-thoracic x-ray irradiation dose of 20 Gy (*n* = 5 per treatment per group) (Figure 5A). The first dose was administered 1 hour before radiation. Upon histological examination 2 weeks after irradiation, there was some protection with anti-CCL22 treatment (Figure 5B), but to a lesser extent than with TLY012 after quantification of inflammation (Figure 5C). Further investigation needs to determine relationships between the TRAIL/DR5 pathway and CCL22 suppression.

### In vivo micro-CT scans of mouse lung show rescue from radiation-induced lung injury and fibrosis following TLY012 treatment.

**Figure 6:**
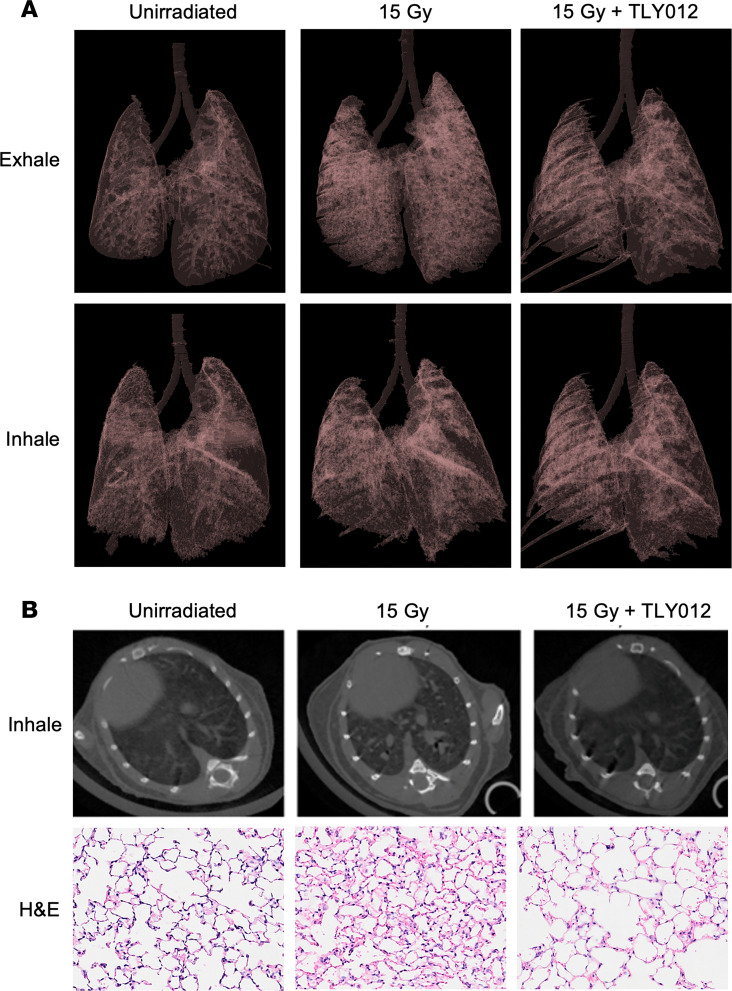
Respiration-gated imaging in mice irradiated with a whole-thorax x-ray irradiation dose of 15 Gy with or without TLY012 treatment. (**A**) 3D reconstruction of mouse lungs from μCT images during exhale and inhale portions of the breathing cycle. Images were subjected to 7% opacity filter in CTVol software. (**B**) Representative μCT images of female *Trail^–/–^* mouse lungs unirradiated, irradiated with 15 Gy, or irradiated with 15 Gy and rescued with TLY012 treatment during inhale duration of the breathing cycle (*n* = 2 per group). Mouse weights were 19.6 g, 24.1 g, and 22.4 g, respectively. H&E slides of lungs reinflated postmortem were prepared. Original magnification, ×10.

To determine radiation effects in vivo, micro–computed tomography (μCT) scans were taken of *Trail^–/–^* female mice unirradiated and 2 weeks after a single thoracic irradiation dose of 15 Gy with or without twice-weekly TLY012 treatment (*n* = 2 per treatment per group). Individual μCT image slices (Supplemental Figure 7A) were collected along with 3D reconstruction of lungs during inhale and exhale duration (Figure 6A) of the breathing cycle. In μCT, mouse lungs expanded more upon inhalation without irradiation and after 15 Gy radiation plus TLY012 treatment compared with 15 Gy controls. The distance between the heart and esophagus was visually greater in maximum-inhale images in the TLY012-treated group versus the 15 Gy control group, demonstrating greater lung expansion (Figure 6B). With 3D reconstruction (Supplemental Table 3), when all images were subjected to the same opacity conditions, unirradiated controls and 15 Gy plus TLY012 lungs were overall clearer and had more visible airway space, particularly during the exhale duration (Figure 6A). These observations are consistent with postmortem findings through H&E staining and immunohistochemistry.

The same experiment was repeated in female *Trail^–/–^* mice, in which mice either were unirradiated controls or received a single whole-thorax x-ray irradiation dose of 20 Gy with or without twice-weekly TLY012 treatment for 2 weeks (*n* = 3 per treatment per group). μCT image slices were consistent with the original experiment, showing decreased lung volume in the inhale portion of the breathing cycle in 20 Gy control mice versus unirradiated control or 20 Gy plus TLY012 mice (Supplemental Figure 7B). There were some abnormalities in the μCT images in the 20-Gy-irradiated control mice around the heart that may represent atelectasis or opaque infiltration. 3D reconstruction of μCT images showed that the 20 Gy control mice had more congested lungs with less visible airway space compared with 20 Gy plus TLY012 or unirradiated mice (Supplemental Figure 7C).

### Preliminary compliance measurements show partial benefit of TLY012 treatment to pressure-volume loop in female Trail^–/–^ mice.

Compliance and pressure-volume (PV) loops were recorded for male and female *Trail^–/–^* mice that received a single whole-thorax x-ray irradiation dose and were treated with TLY012 or remained control (*n* = 1–3 per treatment per group). At 2 weeks after irradiation, there was a slight difference in compliance in both female and male irradiated control and TLY012-treated groups (Supplemental Figure 8, A and B). There was a slight increase in PV loop in female *Trail^–/–^* mice treated with TLY012 compared with the control group (Supplemental Figure 8C). There was almost no change in PV loop in male mice (Supplemental Figure 8D). When the females and males were combined for the control and TLY012-treated groups, a small difference in compliance and PV loop measurements was observed (Supplemental Figure 8, E and F). 19. There were no statistical differences in compliance and PV loop seen in this study. The females treated with TLY012 showed slight improvement in compliance and PV loop, but mouse numbers were limited to 3 females per group. When groups were separated by sex (*n* = 1–3 per treatment per sex), improvement in compliance and PV loop was observed in female 20 Gy plus TLY012 mice compared with female mice that received radiation alone. If groups had contained more than 3 females per group, a greater improvement in lung compliance might have been observed. Thus, while observed trends are promising, larger studies in the future would better assess the impact on lung compliance measurements.

### DR5 agonist and delayed TLY012 treatment show minimal but promising efficacy in rescue from radiation pneumonitis.

We conducted experiments in which TLY012 was administered at 24 and 48 hours after radiation in male *Trail^–/–^* mice that received a single dose of 20 Gy (*n* = 6 per treatment per group) and female C57BL/6 mice that received a single dose of 12 Gy (*n* = 5 per treatment per group), respectively. These experiments demonstrated that time of TLY012 dosing impacts extent of rescue from lung injury. TLY012 administration 24 hours after irradiation showed less rescue than experiments in which the first dose was administered 1 hour before irradiation (Supplemental Figure 9, A and B).

A blinded pathologist assigned an inflammation score based on criteria defined in Gori et al. (56) and determined that mice treated with TLY012 at 24 hours after irradiation generally had a lower inflammatory score compared with controls. To monitor toxicity, complete body weights were recorded twice weekly for the 2-week experiment (Supplemental Figure 9C). A more complete rescue of the lungs was observed in mice given TLY012 one hour before radiation compared with mice that received the drug 48 hours after radiation (Supplemental Figure 9D). Mice were weighed twice a week, and both treated and control mice stabilized after day 8 (Supplemental Figure 9E). While the treatment 48 hours after radiation did not show as much rescue as what was observed with same-day TRAIL, further investigation is needed with post-radiation rescue.

We used the DR5 agonist MD5-1 to investigate the degree of rescue in irradiated mice in comparison with TLY012. Male *Trail^–/–^* mice were irradiated with 20 Gy and then treated with MD5-1 twice weekly or given an isotype control. While MD5-1 provided some rescue, there were many areas of inflamed tissue throughout the lungs. The results were comparable to those seen with the IgG isotype control, implying that the drug did not provide significant rescue in the preliminary experiment without further optimization (Supplemental Figure 9F). Mice were weighed twice a week, and both the IgG isotype control–treated and the MD5-1–treated group recovered on day 8 after radiation (Supplemental Figure 9G). The greatest rescue was observed when TLY012 was administered to the mice 1 hour before radiation.

### Improved survival of mice treated with ONC212 following 20 Gy thoracic irradiation in C57BL/6 mice.

**Figure 7:**
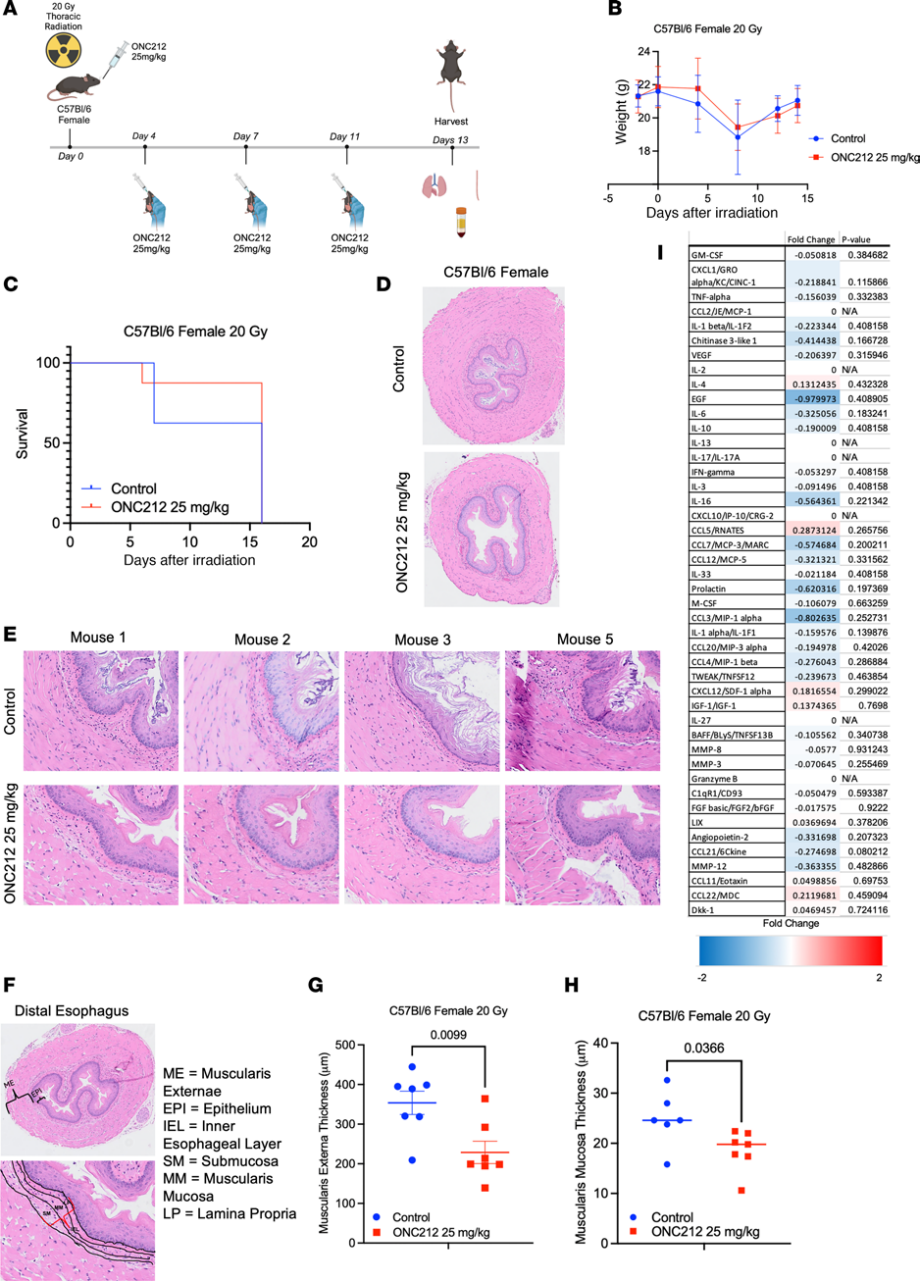
Reduced severity of radiation esophagitis with ONC212 for 2 weeks after a toxic thoracic x-ray irradiation dose in mice. (**A**) Timeline of female C57BL/6 mice irradiated with a single whole-thorax dose of 20 Gy followed by treatment of either control gavage or 25 mg/kg of ONC212 (*n* = 8 per treatment per group). (Created in BioRender. Strandberg J. 2024. https://BioRender.com/z31h016.) (**B**) Weights of mice before radiation and 2 weeks after irradiation up until sacrifice at day 13 (*n* = 8 per treatment per group). (**C**) Kaplan-Meier curve of mice after 20 Gy thoracic irradiation treated with control gavage or 25 mg/kg of ONC212 (*n* = 8 mice per treatment per group). Mice were sacrificed before harvest date for greater than 20% weight loss. (**D** and **E**) Representative H&E images of cross section of distal esophagus of mice treated with control gavage or 25 mg/kg of ONC212 after 20 Gy thoracic irradiation at ×10 (**D**) and ×40 (**E**) original magnification. (**F**) Representative image of cross section of esophagus at ×10 original magnification highlighting tissue layers examined. (**G** and **H**) Quantification of thickness (μm) of different esophageal layers was determined by measurement of 5 random areas around the circumference of the esophagus for each mouse. There was a significant decrease in thickness of the muscularis externa (**G**) and muscularis mucosa (**H**) layers in mice treated with ONC212 (*P* = 0.0099, 0.0366, respectively). (**I**) Heatmap of cytokine fold change between control and ONC212-treated mice (*n* = 8 mice per treatment per group).

To test whether a more potent analog of the TRAIL pathway agonist ONC201 could rescue lethality of thoracic irradiation, we treated a cohort of female C57BL/6 mice with 20 Gy thoracic x-ray radiation followed by 25 mg/kg of ONC212 orally twice a week (Figure 7A). While the weight of the mice decreased slightly at 1 week after irradiation, it appeared to recover by the second week in both control and treated mice (Figure 7B). Mice treated with 20 Gy plus ONC212 had higher survival at 2 weeks after irradiation compared with 20 Gy control mice (Figure 7C).

### Morphological and quantitative evidence of reduced severity of esophagitis following 20 Gy thoracic irradiation among ONC212-treated mice.

Histological sections of esophageal tissue from 20 Gy control mice compared with 20 Gy plus ONC21 mice showed reduced severity of radiation esophagitis (Figure 7). Microscopic examination of distal esophagus from 20 Gy control mice compared with 20 Gy plus ONC212 mice revealed morphological evidence of reduced severity of radiation esophagitis at low or high power (Figure 7, D and E). Quantification of the muscularis externa thickness revealed a significant decrease in mice treated with ONC212 (*P* = 0.0099) (Figure 7, F and G). The muscularis mucosa thickness was also significantly decreased in ONC212-treated mice (*P* = 0.0366) (Figure 7H).

### Cytokine alterations reveal effects of ONC212 that are associated with reduced severity of radiation esophagitis.

Altered levels of cytokines were associated with reduced severity of radiation esophagitis in ONC212-treated mice (Figure 7I). While there was no statistical difference in cytokine expression between control and ONC212-treated mice, there was downregulation of EGF, IL-16, CCL7, prolactin, and CCL3. There was an increase in expression of CCL5, CXCL12, CCL22, and IGF-1.

## Discussion

This work demonstrates prevention of lethality and other severe adverse consequences of therapeutic or unanticipated radiation injury such as pneumonitis, fibrosis, dermatitis, and esophagitis, by short-term treatment with innate immune TRAIL pathway agonists. Although we initially discovered the connection between the TRAIL death receptor signaling pathway and radiation pneumonitis nearly 2 decades ago (57), not until recently was it feasible to test whether this could be addressed through pharmacological interventions. It is surprising that short-term treatment with either TRAIL pathway agonist TLY012 or TIC10/ONC201 for 2 weeks prevents pneumonitis or lethality. This has implications for management of lung toxicity from radiation or as an approach for radiation countermeasures. Prevention of acute lung inflammation prevents delayed effects of radiation on the lungs and chronic lung injury. Similarly, the reduction of dermatitis and esophagitis has important implications for patients receiving radiotherapy.

The TRAIL pathway agonist ONC212 provides an orally bioavailable drug for treating severe esophagitis caused by radiation therapy commonly used in the treatment of lung, breast, esophageal, oral, or head and neck cancers. We restricted our studies to wild-type mice as we would not expect rescue from radiation esophagitis of either *Trail^–/–^* or *Dr5^–/–^* mice with imipridones such as ONC212 based on results from radiation pneumonitis.

The initial radiation dose of a single thoracic x-ray dose of 20 Gy was decided based on Dabjan et al. (58). The review summarized findings from more than 300 research papers that used whole-thorax irradiation across different murine strains. Our study was originally going to be a long-term survival study lasting 24 weeks. The review by Dabjan et al. determined that the radiation dose used to create a median survival time of 24 weeks in C57BL/6 female mice was 20 Gy. We tested the dose of 20 Gy and examined its effects on mice and their lungs at 2 weeks and discovered visible differences in inflammatory response.

A limitation of this study is that the lungs were not reinflated postmortem. We conducted a separate study comparing the H&E staining of inflated lungs versus non-inflated lungs of unirradiated mice and concluded that the alveolar border thickness was not greatly impacted by inflation (Supplemental Figure 1H). Consistently across all our models, the lungs were not inflated, but lung inflammation and its rescue were obvious and were functionally documented in vivo. Another limitation is the number of mice used in each experiment. While individual experiments included a relatively small number of mice, we continued to observe that TLY012 significantly mitigated radiation pneumonitis across multiple experiments.

While there were statistically significant changes in lung tissue mRNA levels of genes related to immune response, there was no statistically significant fold change in inflammatory markers in serum. The lack of significance in cytokines could be because cytokine levels were measured from serum and not from bronchoalveolar lavage (BAL). The mRNA was extracted from lung tissue, while cytokine levels were measured from serum. In future experiments BAL could be used to determine whether differential cytokine activity is more prominent in the lungs. It should be noted that serum samples were taken 3 days after the last administration of TLY012.

There was no difference in DNA damage (γ-H2AX) or T cell inflammation (CD3ε) in irradiated lungs in healthy or inflamed areas of the lungs regardless of whether they were rescued by TRAIL pathway activation. However, rescued mice had far fewer areas of pneumonitis, physiologically relevant increase in oxygen saturation, and evidence of rescued alveolar border thickness. The prevention of pneumonitis by the TRAIL innate immune pathway agonists has no detrimental effect on the antitumor efficacy of therapeutic radiation.

We showed potential for TLY012 to prevent long-term radiation-induced lung fibrosis (RILF). RILF is characterized by accumulation of fibroblasts, myofibroblasts, and ECM proteins such as collagen (59). RILF includes alveolar wall and bronchiolar epithelium thickening via scar tissue (60, 61), which is shown to spread outward into the lung originating from the airways (62). Through our trichrome staining on *Trail^–/–^* mice 22 weeks after 20 Gy thoracic irradiation, we showed that the controls experienced this thickening of bronchiolar epithelium through collagen deposition, while mice that were treated with TLY012 appeared to have more normal lung architecture.

We did not observe any negative side effects with TLY012 treatment, while other DR5 agonists such as MD5-1 have been shown to cause gastrointestinal toxicity as we previously described (63).

Our results provide important clues as to alterations in cytokine biomarkers such as CCL22 or others that are impacted by TLY012. The macrophage-derived chemokine CCL22 has been recognized as a type 2 T helper cell (Th2) chemokine, and its involvement in allergic diseases, such as atopic dermatitis, bronchial asthma, and eosinophilic pneumonia, has been revealed (64, 65). CCL22 is overexpressed in a mouse model of bleomycin-induced pulmonary fibrosis (66). There is selective upregulation of CCL22 in a rat model of radiation pneumonitis (55). CCL22 is localized primarily to alveolar macrophages. It has also been observed in elevated levels in BAL fluid of idiopathic pulmonary fibrosis (IPF) patients. Th2-derived cytokines play pivotal roles in the pathogenesis of IPF (67, 68). Type 2 responses assist with the resolution of cell-mediated inflammation. Our results point to a previously unknown approach to treat the acute effects of severe radiation toxicity to the lungs by targeting CCL22 alone or in combination with blockade of other mediators such as TGF-β or suppressors such as TRAIL pathway agonists.

While sex differences in response to radiation and other immune diseases are not well understood, they are thought to be the result of a gene dosage effect. The X chromosome contains many genes involved in innate immunity, such as *TLR7*, which encodes Toll-like receptor 7 (TLR7). Approximately 23% of X-linked genes escape deactivation, including those that impact immune function (69). We confirmed this by finding a significant increase in TLR7 expression in female *Trail^–/–^* mice 2 weeks after irradiation with 20 Gy. Further investigation is needed to elucidate the mechanism. Other studies have found that, after radiation, female mice develop injuries more quickly and have more positive staining for fibrotic markers and, overall, more severe fibrosis compared with males (70).

The results regarding the decrease in skin fibrosis are comparable to those of Park et al., who observed reversal of skin fibrosis in mouse models of scleroderma after treatment with TLY012 (43). In their study, mice were injected with bleomycin, an agent known to cause an inflammatory response, and 6 weeks later were treated with TLY012 for 3 weeks before the skin was analyzed. Their results showed that dermal myofibroblasts upregulated DR5 and became sensitive to apoptosis through TLY012. They also demonstrated the antifibrotic efficacy of TLY012 in a mouse model of scleroderma, known as tight-skin-1 (*Tsk-1*) mice, as after treatment there was a decrease in hypodermal thickening, collagen deposition, and myofibroblast accumulation. Our results showing a decrease in radiation-induced dermatitis after treatment with TLY012 demonstrate the capability of broader skin fibrosis modulation by TRAIL/DR5 pathway agonists.

While H&E-stained images of lungs showed reduced inflammation in mice treated with TLY012, changes in gene and cytokine expression showed a decrease in inflammation as well. Genes related to a decrease in inflammatory response are increased in TLY012-treated mice. Killer cell lectin-like receptor, subfamily A, member 7 (KLRA7) is expressed by mature NK cells in mice (71). RelB, a member of the NF-κB/RelB family, acts as a transcription suppressor in fibroblasts that limits the expression of proinflammatory mediators, which were increased in TLY012-treated mice (72). Transmembrane protein 173 (TMEM173) encodes the protein stimulator of interferon genes (STING), a key mediator in host defense against damaged cells. TMEM173 plays an important role in normal pulmonary function, as loss-of-function TMEM173 alleles in humans lead to pulmonary fibrosis (73). HERC6 mediates ISGylation, involved in DNA repair and autophagy (74, 75). IL-1 receptor like 2 (IL1RL2), which has antiinflammatory effects and regulates macrophage function, is upregulated in mice treated with TLY012 (76). These findings suggest that TLY012 can prevent inflammation and fibrotic effects caused by radiation.

Decreased expression of genes related to a proinflammatory response are also observed in TLY012-treated mice. Dedicator of cytokinesis 9 (*Dock9*), which plays a role in immune disorders, showed a decrease in mRNA expression in TLY012-treated mice (77). Mitogen-activated protein kinase 8 (*Mapk8*), also known as c-Jun amino-terminal kinase (*Jnk*), which is activated in response to various cellular stresses and promotes production of proinflammatory and profibrotic molecules, is significantly decreased in TLY012-treated mice (78). Prostaglandin-endoperoxide synthase 2 (*Ptgs2*), also known as Cox2, causes chronic inflammation (79). Retinoic acid early transcript 1α (*Raet1A*), which functions as a stress-induced ligand for NKG2D receptor expressed on cytotoxic immune cells, is downregulated in TLY012-treated mice, suggesting the control mice are in a greater state of stress (80). Another indicator of inflammation, B cell leukemia/lymphoma 6 (*Bcl6*), is decreased in treated mice (81).

Reduced severity of esophagitis was associated with downregulation of EGF, IL-16, CCL3, CCL7, and prolactin, suggesting decreased inflammation. EGF is involved in regulation of epithelial cell proliferation, growth, and migration. IL-16 is a chemoattractant for peripheral immune cells linked to several inflammatory diseases (82). CCL3 is associated with macrophage recruitment and contributes to radiation-induced injury (83). CCL7 is associated with monocytes and is secreted by stressed epithelial cells, thus recruiting macrophages (84). Prolactin is involved in immune response and is upregulated in inflammatory esophageal conditions such as gastroesophageal reflux disease (85). Upregulation of CCL5, CXCL12, CCL22, and IGF-1 shows a trend toward healing radiation damage. While CCL5 has been shown to contribute to radiation-induced injury, it has also been shown to protect normal tissue as a hematopoietic growth factor and could promote hematopoietic cell cycling. CCL22 is involved in immune regulation by promoting regulatory T cell communication with dendritic cells in lymph nodes (86). Treatment with IGF-1 restored salivary gland function in irradiated mice (87). Although there were some changes in cytokine expression, a statistically significant difference was not observed, possibly because serum samples were taken on day 13 after irradiation. Inflammation may have started to subside with some healing, and so at earlier time points there may have been a greater fold change.

For esophagitis rescue by ONC212, we did not evaluate combination of chemotherapy such as 5-fluorouracil, cisplatin, or oxaliplatin with radiation as occurs for patients receiving concurrent chemoradiotherapy. We used a single high dose of thoracic x-ray irradiation in mice rather than fractionated radiotherapy as with human patients, and we investigated thoracic radiation rather than esophageal radiation with lung shielding. Thus, while there is reduced esophagitis with ONC212 treatment (Figure 7), improved survival in Figure 7C may be due to rescue from radiation pneumonitis as well as esophagitis. Additional future directions include studies of other TRAIL pathway agonists such as TRAIL, TLY012, or other imipridones, including ONC201 or ONC206, to mitigate the severity of radiation esophagitis.

Research directions of interest include further mechanistic studies on cytokines and chemokines in the pathogenesis of pneumonitis or its rescue. Questions arise regarding crosstalk between TRAIL and TGF-β pathways, Gas/STING, and other cytokines linked to radiation pneumonitis. Our findings have relevance to other lung injury models such as adult respiratory distress syndrome, IPF, immune checkpoint blockade pneumonitis, and COVID-19 pneumonitis and to toxicities of chemotherapy such as bleomycin. Combinations with steroids or TGF-β pathway inhibitors and CCL22 blockade need to be investigated, as do differences between males and females in how they develop lung inflammation and fibrosis. While our results point to TLR7, more studies are needed to further unravel underlying differences in more severe phenotypes in female *Dr5^–/–^* and *Trail^–/–^* mice.

Further studies can investigate protection to other organs, including bone marrow, gastrointestinal tract, or brain, from toxic effects of radiation. It remains to be determined whether TRAIL pathway agonists can impact on various forms of dermatitis and their underlying mechanisms. More work is needed to determine how late in time administration of TRAIL pathway agonists could impact on protection from radiation after exposure, including in combination with other agents. The strategy to reduce radiation esophagitis merits further investigation given the morbidity patients face following therapeutic radiotherapy.

Our results suggest that some rescue is possible even at 48 hours after irradiation and treatment by TLY012 (Supplemental Figure 9). Our findings have translational relevance by suggesting future clinical investigation of the TRAIL innate immune pathway, CCL22, and TLR7 in toxicity of radiation or other cancer therapeutics and in other inflammatory lung or skin conditions, and have implications for radiation countermeasures.

## Methods

Further information can be found in Supplemental Methods.

### Bioassays

#### Sex as a biological variable.

All studies separated mouse groups based on sex, as males and females have different immune responses to radiation. While both males and females develop adverse responses to high doses of radiation, we studied sex as a variable.

#### Genetically modified mice.

Mice with null mutations of DR5 (*Tnfrsf10b^–/–^*) were generated and characterized by our laboratory (39, 40). *Tnfsf10tm1b-*KO sperm was obtained from the UC Davis Mouse Biology Program and crossed with C57BL/6 females (Taconic) until a homozygous *Trail^–/–^* background colony was obtained. Both strains were backcrossed for 1 generation onto a C57BL/6 background.

Animal studies were carried out at Brown University facilities with approval from the Brown University IACUC. Wild-type C57BL/6 mice (Taconic), *Dr5^–/–^*, and *Trail^–/–^* were given a single whole-thorax x-ray (Philips RT250) irradiation dose of 20 Gy with shielding of other organs. Eight- to fifteen-week-old male and female mice of each genotype were used. Starting 1 hour before irradiation, mice were treated with either 10 mg/kg of TLY012 (D&D Pharmatech) in Dulbecco’s phosphate-buffered saline (PBS) solution (Cytiva SH30264.02) by intraperitoneal (i.p.) injection, 100 mg/kg of ONC201/TIC10 (Chimerix/Oncoceutics) by oral gavage (p.o.), or a control gavage consisting of 20% Cremophor EL (Sigma-Aldrich 238470) and 70% PBS per volume p.o. (*n* = 2 per genotype per sex per treatment). Treatment was continued for 2 weeks, with TLY012 administered twice weekly and ONC201 administered once a week. Mice were euthanized 13 days after irradiation. Experiments using TLY012 treatment in female C57BL/6, *Dr5^–/–^*, and *Trail^–/–^* mice were repeated in a larger cohort size (*n* = 8–10 mice per treatment per group).

For esophagitis studies, 11-week-old female C57BL6 (Taconic) mice were given a single whole-thorax x-ray irradiation dose of 20 Gy with shielding of other organs. Mice were separated into either control p.o. or 25 mg/kg of ONC212 p.o. twice weekly for 2 weeks (*n* = 8 mice per treatment per group). Mice received their first treatment 1 hour before radiation was administered. Mice were sacrificed on day 13 after irradiation, or earlier if greater than 20% weight loss was observed. SPOT software (SPOT Imaging) was used to measure width of muscularis externa (ME) and muscularis mucosa (MM) of distal esophagus. Five random measurements around the circumference of the esophagus were made for both ME and MM and then averaged together for each mouse.

For pneumonitis and esophagitis experiments, organs were harvested from mice, and 600 μL of blood was collected via cardiac puncture for serum cytokine analysis. Complete blood count was measured with a HEMAVET 950FS (Drew Scientific), which was calibrated for murine blood samples. Organs were preserved in 10% formalin, transferred to 70% ethanol, embedded in paraffin, and sectioned at 5 μm. H&E-stained slides of the lungs, heart, liver, and duodenum were imaged at ×4 and ×40 magnifications on a Nikon Y-THM Multiview Main Teaching Unit microscope using a Diagnostic Instruments Inc. model 18.2 color mosaic camera with SPOT Basic version 5.3.5 software.

H&E images of lungs were given an inflammatory score based on five to ten ×200 fields with obvious alveolar thickening by a blinded pathologist using a modified scoring system based on Gori et al. (56). An interstitial cell count per 0.25 mm^2^ based on an area of higher concentration of cellular infiltrates and lesions was provided for each mouse lung.

Survival experiments were conducted using male *Trail^–/–^* mice treated with TLY012 or control exposed to a single whole-thorax radiation dose of 18 Gy (*n* = 5 per treatment), as any dose above 18 Gy caused radiation toxicity too great to allow long-term survival (Supplemental Figure 3A). *Trail^–/–^* female mice were also subjected to survival study with a single whole-thorax radiation dose of 15 Gy or 18 Gy and treated with the same regimen of TLY012 or control (*n* = 2 per radiation dose per treatment) (Supplemental Figure 3B). To determine lethal dose of radiation for C57BL/6 mice, female C57BL/6 mice were given a single dose of whole-thorax irradiation of 25 Gy in a survival study in which they either were treated with TLY012 or remained control (*n* = 6 per treatment) (Supplemental Figure 3C).

### Tumor-bearing model

C57BL/6 female mice (Taconic) were injected with 100,000 cells of murine breast cancer (e0771) in the right second mammary fat pad on day 0 of experiment. The e0771 cell line is classified as Luminal B, ERα^–^, ERβ^+^, PR^+^, and Her2^+^ (ATCC; pathogen-tested at Charles River Laboratories) (88). On day 9, tumors were greater than 4 mm in diameter, and mice were grouped into control or treatment with 10 mg/kg of TLY012 twice weekly, 100 mg/kg of ONC201 once a week, or a combination of both drugs. Each group (*n* = 3–4 per treatment per group) also received a single whole-thorax x-ray irradiation dose of 20 Gy with shielding of other organs.

One group (*n* = 3) received no treatment or radiation. The long axis and short axis of the tumors were measured using calipers and recorded twice weekly. On day 18 mice were euthanized at a humane endpoint, defined by 20% weight loss and/or a tumor volume of 2,000 mm^3^ (~10% of body weight). Once extracted, tumors were weighed and measured to determine final volume and weight.

Pulse oximeter readings were taken initially before radiation treatment and before sacrifice 9 days after radiation (MouseSTAT Jr. Pulse Oximeter, Kent Scientific). When mice were under anesthesia, the sensor of the MouseSTAT Jr. was placed on the hind right paw for 30 seconds. The highest and lowest values shown by the pulse oximeter during this time were recorded and then averaged together. The mean of all the averages per treatment group was then calculated.

### Anti-CCL22 antibody treatment

Seven-week-old *Trail^–/–^* female mice received a single thoracic x-ray irradiation dose of 20 Gy with shielding of other organs and were treated with either 20 μg of anti-CCL22 antibody (AF439, R&D Systems Inc.) dissolved in 500 μL of sterile normal saline via i.p. injection every other day for 2 weeks or 20 μg of polyclonal goat IgG (AB-108-C, R&D Systems Inc.) dissolved in 500 μL of normal saline via i.p. injection every other day for 2 weeks (*n* = 5 per treatment per group). Treatment began 1 hour before radiation, and mice were weighed twice weekly. On day 13 after irradiation, mice were sacrificed.

### Cytokines

Mouse serum samples were analyzed by a custom Murine Premixed Multi-Analyte Kit (R&D Systems Inc.) using a Luminex 200 Instrument (Luminex Corp.) according to the manufacturer’s instructions. Murine serum levels of angiopoietin-2, BAFF/BLyS/TNFSF13B, CCL2/JE/MCP-1, CCL3/MIP-1α, CCL4/MIP-1β, CCL5/RANTES, CCL7/MCP-3/MARC, CCL11/eotaxin, CCL12/MCP-5, CCL20/MIP-3α, CCL21/6Ckine, CCL22/MDC, chitinase 3–like 1, CXCL1/GROα/KC/CINC-1, CXCL10/IP-10/CRG-2, CXCL12/SDF-1α, Dkk-1, FGF-basic/FGF-2/bFGF, GDF-15, GM-CSF, granzyme B, IFN-γ, IGF-I/IGF-1, IL-1α/IL-1F1, IL-1β/IL-1F2, IL-2, IL-3, IL-4, IL-6, IL-7, IL-10, IL-13, IL-16, IL-17/IL-17A, IL-27, IL-33, M-CSF, MMP-3, MMP-8, MMP-12, prolactin, TWEAK/TNFSF12, VEGF, and VEGFR2/KDR/Flk-1 were measured. Analyte values were reported in picograms per milliliter (pg/mL).

### Immunohistochemistry

Formalin-fixed paraffin-embedded sections of tissue were deparaffinized in xylene and rehydrated through graded ethanol solutions to PBS. Heat-induced antigen retrieval (in 0.01 M citrate buffer; pH 6.0) and endogenous peroxidase blocking (3% H2 O2) were done using a standard protocol. Diluted primary antibody (Cell Signaling Technology [CST] 8242, 1:800; CST 99940, 1:150; CST 9718, 1:500; Abcam ab107099, 1:100; Leica P53-CM5P-L, 1:200; Novus Biologicals NBP2-24906, 1:200; Novus Biologicals NB100-56116SS, 1:2,000; Novus Biologicals NB100-56113, 1:1,000) was applied, and slides were incubated at 4°C overnight. Slides were washed with PBS and incubated at room temperature for 1 hour with secondary antibodies (Vector Laboratories MP-7401, MP-7402). Slides were developed with diaminobenzidine substrate (Vector Laboratories SK-4100) and counterstained with hematoxylin (Richard Allen Scientific). Slides were scanned (Olympus VS200), and representative bright-field images were taken and quantified with QuPath v0.5.1 software (University of Edinburgh, Edinburgh, United Kingdom).

### In vivo micro-CT imaging

For micro-CT (μCT) imaging, mice were imaged in a SkyScan 1276 in vivo μCT scanner (Bruker) under isoflurane at 41 μm voxels with a 0.7° rotation step in supine position. Mice that underwent μCT of lungs comprised *Trail^–/–^* females that were unirradiated and irradiated at 15 Gy with or without 10 mg/kg of TLY012 twice a week (*n* = 2 per group). Mice underwent μCT scans at 13 days after irradiation. Image-based respiratory gating was used to sort images into gates based on specific time points of the breathing cycle to minimize motion artifacts in final reconstructions. For 3D reconstruction, Dataviewer version 1.5.4 (Bruker) was used to sort CT images using the function Listmode Scan grouping images in each bin where bin 0 = maximum exhale and bin 3 = maximum inhale (empty views <10%). Once images were sorted, the listmode dataset was opened in NRecon software (Bruker), where regions of interest underwent 3D reconstruction. Reconstructed images were analyzed in CTAn software (Bruker), where lung volumes were calculated during maximum inhale and maximum exhale. The plug-ins in the custom processing page were used to create a region of interest that separated lungs from the rest of the image. 3D reconstructed images of lung only were opened in CTVol software (Bruker), where color (red, 69%; green, 50%; blue, 50%) and opacity (7%) of lung were changed (Figure 6A). H&E-stained slides were prepared postmortem after lung reinflation with 1 mL of PBS and sectioning (Figure 6B).

The experiment was repeated with female *Trail^–/–^* mice, in which mice either were unirradiated controls or received a single whole-thorax x-ray irradiation dose of 20 Gy and received either no treatment or TLY012 twice weekly for 2 weeks (*n* = 3 per treatment per group). Two weeks after irradiation, mice underwent μCT scans and then were euthanized. μCT scans underwent 3D reconstruction for the inhale portion of the breathing cycle and were compared across treatment groups (Supplemental Figure 7, B and C).

### Statistics

Statistical analyses for inflammatory scores and interstitial cell counts between control and TLY012-treated mice in C57BL/6, *Dr5^–/–^*, and *Trail^–/–^* mice were determined using 1-tailed Mann-Whitney test. Comparison of cytokines between treatment groups was determined by 1-way ANOVA with post hoc Tukey’s tests for pairwise comparisons on R software. Immunohistochemical (IHC) quantification was analyzed using 2-tailed Student’s *t* tests. IHC TLR7 comparison was analyzed using 1-way ANOVA with post hoc Tukey’s tests for pairwise comparison. Complete blood count data were analyzed using multiple *t* tests corrected for FDR with the Benjamini-Yekutieli method. Mouse data are expressed as mean, and error bars represent ± SEM. *P* values ≤ 0.05 were considered significant. Statistical analysis and graphs were performed using R and GraphPad (GraphPad Software Inc.).

### Study approval

Animal studies were approved by the Institutional Animal Care and Use Committee (IACUC) of Brown University in accordance with federal guidelines. Brown University IACUC Protocol 22-02-0004 covering the included studies was initially approved on January 15, 2020, and reapproved on April 8, 2022 (expires April 7, 2025).

### Data availability

Data are available in the Supporting Data Values file or associated supplemental files. NanoString raw data files are accessible through the NCBI’s Gene Expression Omnibus (GEO) series (accession number GSE278401).

## Author contributions

WSED conceptualized the study. WSED, JS, AL, MH, PS, AG, ADLC, PDLC, L Zhang, LHB, KEH, AJKS, FT, and L Zhou established methodology. WSED, JS, AL, MH, PS, AG, ADLC, PDLC, L Zhang, LHB, KEH, AJKS, FT, and L Zhou performed investigations. WSED, JS, AL, MH, PS, AG, ADLC, PDLC, L Zhang, LHB, KEH, AJKS, FT, and L Zhou performed visualization. WSED and AEA acquired funding. SL provided funding and TLY012. PPK, DEW, AAS, TAD, SLG, CGA, SIR, and EY provided field expertise and feedback. WSED performed project administration. WSED supervised the study. WSED wrote the original draft of the manuscript. All authors reviewed and edited the manuscript.

## Supplementary Material

Supplemental data

Supporting data values

## Version 1 01/14/2025

In-Press Preview

## Version 2 03/03/2025

Electronic publication. Includes correct version of Supplemental Figure 7C.
