# Supplementary material for: TRAIL agonists rescue mice from radiation-induced lung, skin, or esophageal injury
Source: J Clin Invest. 2025 Jan 14;135(5):e173649. doi: 10.1172/JCI173649 (PMC11870730; doi:10.1172/JCI173649)
Supplement: Supplemental data [file jci-135-173649-s156.pdf]

Methods

Bone Marrow Preparation

Whole sternum was harvested for histological analysis of bone-marrow. The sternum was placed in 10% formalin for 24-hours and then transferred to 10:1 volume-to-volume ratio of fixative to sample of ethylene diamine tetra acetic acid (EDTA) (pH 8) at room temperature for 2-weeks for decalcification. The EDTA was refreshed once a week for two-weeks and then the sternum samples were transferred to 70% ethanol prior to paraffin embedding and sectioning. Samples were stained with H&E and cellularity was determined by a pathologist blinded to the study.

TLY012 treatment at 24 and 48-hour post-radiation

Female 8-week-old C57Bl/6 mice (Taconic) were given a single whole-thorax x-ray irradiation dose of 12-Gy with shielding of other organs. At 48-hours after administration of radiation, mice were grouped into control or treated with 10 mg/kg of TLY012 twice weekly (n=4/treatment). Male 15-19 week old *Trail-/-* mice were given a single whole-thorax x-ray irradiation dose of 20-Gy. At 24-hours after radiation, mice were grouped into control or 10 mg/kg of TLY012 twice weekly and received their first treatment (n = 6/treatment/group). Mice were weighed twice weekly, and euthanized on day-13 post-irradiation. Lungs were harvested and preserved in 10% formalin then embedded in paraffin and sectioned at 5 μm thickness. H&E-stained slides of lungs were imaged at 20X magnification.

DR5 agonist

8–13-week-old male *Trail-/-* mice were given a single whole-thorax x-ray irradiation dose of 20-Gy with shielding of other organs. An hour before radiation, mice were treated with 100 μg anti-DR5 mAb (clone MD5-1; BioXCell) or given 100 μg isotype control of polyclonal Armenian hamster IgG (BE0091; BioXcell) via IP injection (n=3/treatment). Mice were treated once a week for two-weeks and sacrificed on day 13-post irradiation. Lungs were harvested and preserved in

10% formalin then embedded in paraffin and sectioned at 5 μm. H&E-stained slides of lungs were imaged at 20X magnifications.

NanoString Assay

Male and female 9-week old *Trail*-/- mice were given a single whole-thorax x-ray irradiation dose of 20-Gy with shielding of other organs. One-hour before radiation mice were grouped into control or given 10 mg/kg of TLY012 via IP injection (n = 3/gender/treatment). Mice were treated and weighed twice weekly up until 11-days post-radiation when all mice were euthanized.

Rodent sacrifice was performed following anesthetization with 100 mg/kg ketamine and 10 mg/kg xylazine administered intraperitoneally. ~400-600 μL blood was collected through cardiac puncture with a 26G 5/8" needle through the intercostal space into the left ventricle. Following cervical dislocation, the inferior vena cava (IVC) and descending abdominal aorta were severed prior to bilateral thoracotomy to expose the heart and lungs. 1 mL of 2 mg/mL EDTA in PBS was injected through the right ventricle to perfuse the lungs, after which the lungs were excised, washed further in PBS, submerged in RNAlater (Sigma-Aldrich, #R0901, Missouri, USA), and flash frozen in liquid nitrogen.

Frozen lung tissue was stored long-term at -80ºC. Thawing was performed over 16-hours at 4ºC. Tissue homogenization was performed with Precellys Lysing Kit (Cayman Chemical Company, #16859, Michigan, USA) and a Bulley Blender Homogenizer (Next Advance, #G14-G15) prior to RNA extraction with QIAgen RNeasy kit (Qiagen, #74104, Hilden, Germany) and cleaned up with QIAgen RNeasy MinElute Cleanup kit (Qiagen, #74204, Hilden, Germany). Quality and concentration were verified by Nanodrop. Extracted RNA samples were gene expression profiled by NanoString nCounter PanCancer Immune Profiling Panel (NanoString Technologies, #XT-CSO-MIP1-12, Seattle, WA) according to the manufacturer's instructions.

Nanostring data was analyzed in nSolver Advanced Analysis Software and ROSALIND. Raw data was uploaded to nSolver for automated normalization, background subtraction, and quality control (QC) check. All samples passed QC. Control mice and mice treated with TLY012 lung samples were used to construct two groups to which an unpaired t-test to generate data in volcano plot created in ROSALIND (**Figure S5)**. Differential expression was determined with p-values and Benjamini-Yekutieli adjusted p-values. Pathway scores are generated in nSolver as a summary of expression level changes of biologically related groups of genes. Pathway scores were derived from first Principal Components Analysis (PCA) scores (1st eigenvectors) for each sample based on individual gene expression levels for measured genes within a specific pathway. The cell-type score was calculated as mean of log2 expression levels for probes included in final calculation for specific cell-type.

Pulmonary Function Testing

Measurements of lung function were obtained by forced maneuvers using flexiVent (SIREQ Scientific Respiratory Equipment Inc., Montreal, QC, Canada) at Yale University. 7-9 week old *Trail-/-* mice (n = 1-3 per treatment/gender/group) were anesthetized with Xylazine (10 mg/kg) and ketamine (100 mg/kg) and Pancuronium bromide (100 µg/kg). Mice were tracheotomized with a 1.2 cm long-metal 18-gauge cannula which then connected mouse to ventilator via Y-tubing. Animals were mechanistically ventilated with tidal volume of 8 ml/kg with frequency of 2.5 Hz using computer-controlled volume ventilator integrated in flexiVent system. Respiratory mechanics were determined by application of predefined pressure/volume perturbations to airways. Compliance and Pressure-volume Loops (PV-loops) were obtained by linear single-compartment model using multiple linear regression. All measurements were carried out until three acceptable readings (coefficient of determination >0.95) were recorded per mouse, and average calculated. After lung function test, all mice were euthanized, and the lung was harvested for further histologic evaluation.

Supplementary Figure Legends

Figure S1. Radiation damage in other organs in the thorax

Lack of toxicity to duodenum **(A),** liver (**B),** and heart **(C)** after radiation (only chest radiation was administered). **D)** No differences in bone marrow cellularity in the sternum of mice 2 weeks post thoracic-irradiation of 20 Gy in mice with or without treatment (40X**, E, F,G)** average of mouse whole body weights for the duration of the two weeks post whole-thorax x-ray irradiation of 20 Gy for C57Bl6, *Dr5-/-,* and *Trail-/-* mice respectively (n = 2/gender/genotype/treatment). Data represents mean ± SD. **H)** Lungs were not reinflated post-mortem as determined by H&E-stained lung tissue at x10 and x20 inflated versus not inflated did not indicate major differences (n = 3 inflated, n = 1 not inflated).

Figure S2. Inflammatory and cytokine alterations after radiation and TRAIL pathway agonist treatment in male and female mice. A,B) Percent inflammation of female C57Bl/6 and *Dr5-/-* mouse lungs determined by whole lung analysis in mice treated with TLY012 or control (n = 7-10/treatment/genotype) **C, D)** Interstitial cell count per 0.25 mm^2 of lung tissue in C57Bl/6 and DR5-/- female mice treated with TLY012 or control (n = 7-10/treatment/group). Cell counts were taken in areas of high cellular infiltration in the lungs (one-tailed Mann-Whitney test). Data represents mean ± SEM. **D)** Heat map of cytokine level fold change 13 days post-irradiation of both male and female mice treated with TLY012 compared to irradiated control of both C57Bl/6 and *Dr5-/-* genotypes (n = 4). **E)** Heat map of cytokine level fold change of mice treated with either TLY012, ONC201, or a combination of both drugs compared to irradiated control 13 days post-irradiation separated by genotype and gender (n = 2).

Figure S3. Protection for chest-irradiation induced mouse lethality by TLY012. A) Survival of male *TRAIL*-/- mice following 18-Gy irradiation led to increased survival in mice treated with 10 mg/kg of TLY012 at 10-weeks following whole-thorax irradiation (n = 5/treatment/group). **B)** Survival of female *TRAIL*-/- mice following 18-Gy whole-thorax irradiation was increased to 3-weeks in mice treated with TLY012 compared to controls that survived 2-weeks post-irradiation (n = 2/treatment/group). **C)** Survival of female C57Bl/6 mice was increased by approximately a week in mice treated with TLY012 compared to controls (n = 6/treatment/group).

Figure S4. Immunohistochemical analysis of lung tissue irradiated with a single dose of 20 Gy x-ray irradiation in mice with C57Bl/6, *Dr5-/-,* and *Trail-/-* genetic backgrounds +/- rescue with TRAIL pathway agonists. A) C57Bl/6 mice given a single whole-thorax irradiation dose of 20 Gy and treated with TLY012, ONC201, or control gavage were euthanized on day 13 post-irradiation. Immunohistochemical analysis of CD3ε was performed to determine T-cell infiltration. **B)** Mice treated with TLY012 showed a decrease in gamma-H2AX in both inflamed and healthy areas of lung tissue (top row x20, bottom row x40). **C)** Immunohistochemistry of female mice shows abundant infiltration of NFkB in all three genotypes C57Bl/6, *Dr5-/-,* and *Trail-/-* in all treatment groups. Scale bar: 100 µm. **D)** Immunohistochemistry shows abundant labeling of the proliferation marker Ki67 (DAB, brown staining) in *Dr5-/-* control mice 13 days-post whole-thorax irradiation of 20 Gy. **E)** Trichrome staining showed an increase in collagen deposition (light blue) in WT and *Trail-/-* control mice compared to those rescued with TRAIL pathway agonist. **F, G, H)** p53 in male and female mice with C57Bl/6, *Dr5-/-,* and *Trail-/-* backgrounds respectively. Areas of inflamed tissue and healthy tissue were examined at x20 **I)** IHC of cleaved caspase-3 and combined quantification **J)** of male and female mice show varied expression in *Trail-/-* mouse lung (n = 16/group). **K)** IHC of cleaved caspase-8 and quantification **L)** of combined *Trail-/-* male and

female lung shows evidence of apoptosis two weeks post irradiation (n = 16/group). Data represent mean ± SEM.

Figure S5. Nanostring nCounter PanCancer Immune panel for *Trail*-/- mice with and without rescue from radiation injury by TRAIL agonist shows immune pathways were significantly impacted by TLY012 treatment A) Volcano plot of all 40 reference genes and their fold-change compared to controls (equal number male and female mice). **B)** Heat-map of log2 fold-change of the top 16 significantly different genes between control and TLY012 treatment (n=6). (1-way ANOVA with Tukey's post hoc test) **C)** Heat-map of serum cytokine level fold-change of TLY012-treated mice relative to untreated controls. **D)** Volcano plot of interferon pathway genes with significant differential expression in the TLY012 treated group compared to control (n = 6/treatment/group). **E)** Heat map of the fold-change of genes in the interferon pathway and their statistical significance. **F)** Volcano plot of antigen processing pathway genes with significant differential expression in the TLY012 treated group compared to the control (n = 6/treatment/group). (1-way ANOVA with Tukey's post hoc test). **G)** Heat map of the fold change of genes in the antigen processing pathway and their statistical significance.

Figure S6. Histological and cytokine alterations in female immune competent C57Bl/6 mice with breast tumor xenograft -/+ rescue with TRAIL pathway agonists. A) Average total body weight of mice from orthotopic tumor injection (day = -9), through radiation (day = 0), up until sacrifice on day 9 post-irradiation (n=3/treatment/group). Data represent mean ± SD **B)** H&E stains of lung tissue 9 days-post irradiation showed a reduction in alveolar-wall thickness and decreased inflammation in mice treated with TLY012. **C)** Heat map of cytokine level fold change of treated mice to unirradiated control.

Figure S7. Additional representative μCT images of mice unirradiated and irradiated with 15 Gy whole-thorax x-ray irradiation and treated with or without TLY012. A) μCT images of the exhale duration of breathing cycle of mice that were unirradiated (top row), received one whole-thorax x-ray irradiation dose of 15 Gy (middle row), and received 15 Gy irradiation in addition to rescue with twice a week treatment of TLY012 for two weeks (bottom row) (n = 2/treatment/group). **B)** μCT images of the inhale duration of the breathing cycle in mice that were unirradiated, received one whole-thorax x-ray irradiation dose of 20 Gy, and received 20 Gy irradiation in addition to rescue with twice a week treatment of TLY012 (n = 3/treatment/group). **C)** 3D reconstruction of the μCT images during exhale and inhale portions of the breathing cycle. All images are subjected to a 7% opacity filter in CTVol software.

Figure S8. Improvements in PV loop of female *Trail-/-* mice treated with TLY012 compared to control mice two weeks after 20 Gy thoracic radiation. A,B) Compliance and **C,D)** PV Loop of female and male *Trail-/-* mice 2 weeks post thoracic x-ray irradiation of 20 Gy (n = 1-3/treatment/group). **E,F)** Combined male and female *Trail-/-* mice compliance and PV loop (n = 3-6/treatment/group). Data represents mean ± SEM.

Figure S9. Additional TRAIL pathway agonist experiments including 24 and 48-hour post-irradiation treatment with TLY012. A) H&E images of lung tissue in male *Trail-/-* mice that had the first dose of TLY012 administered 24-hours post whole-thorax x-ray irradiation of 20 Gy.**B)** Quantification of percent inflammation in the lungs 2 weeks post radiation (n = 6/treatment/group). Data represent mean ± SEM (one-tailed Mann-Whitney test). **B)** Average full body weights of control mice or mice that began twice a week treatment withTLY012 24 hours after receiving one whole-thorax x-ray irradiation dose of 20 Gy (n = 6/treatment/group). **C)** H&E images of female C57Bl/6 mouse lung that received their first dose of TLY012 48-hours post whole-thorax x-ray

irradiation dose of 12 Gy**. D)** Average full body weights of control mice or mice that began twice a week treatment with TLY012 48 hours after receiving one whole-thorax dose of 12 Gy (n = 4/treatment/group) **E)** H&E images of male *Trail-/-* mice given a single whole-thorax x-ray irradiation dose of 20 Gy and then treated with antibody MD5-1 or control IgG isotype once a week. **F)** Whole-body weights of mice treated with MD5-1 or IgG isotype average for two weeks of treatment post-radiation (n = 3/treatment/group). Data represents mean ± SD.

Table S1. CBC of female C57Bl/6 mice at 1 week or 2 weeks post whole thorax x-ray irradiation of 20 Gy. There were no statistical differences between any CBC values of mice irradiated with 20 Gy and then treated with TLY012 or control at one week or two weeks post irradiation (n = 4/treatment/timepoint) (student's t-tests corrected for FDR with Benjamini-Yekutieli method).

	Unit	Control (1 Week)	TLY012 (1 Week)	P-value	Control (2 Week)	TLY012 (2 Week)	P-value
WBC	**K/μL**	**2.74**	**1.41**	**0.051**	**3.40**	**2.74**	**0.251**
NE#	**K/μL**	**0.62**	**0.45**	**0.451**	**1.03**	**0.93**	**0.750**
LY#	**K/μL**	**1.94**	**0.86**	**0.028***	**2.21**	**1.62**	**0.101**
MO#	**K/μL**	**0.17**	**0.09**	**0.116**	**0.13**	**0.13**	**0.887**
EO#	**K/μL**	**0.018**	**0.008**	**0.304**	**0.015**	**0.043**	**0.448**
NE%	**%**	**22.52**	**27.82**	**0.541**	**30.79**	**31.06**	**0.970**
LY%	**%**	**70.77**	**63.70**	**0.365**	**64.60**	**62.06**	**0.746**
MO%	**%**	**6.04**	**6.98**	**0.576**	**3.90**	**4.94**	**0.499**
EO%	**%**	**0.49**	**1.09**	**0.457**	**0.53**	**1.37**	**0.441**
BA%	**%**	**0.18**	**0.41**	**0.378**	**0.18**	**0.58**	**0.444**
RBC	**M/μL**	**10.68**	**8.47**	**0.201**	**9.07**	**8.23**	**0.461**

HB	**g/dL**	**15.80**	**12.73**	**0.215**	**13.23**	**12.50**	**0.658**
HCT	**%**	**53.73**	**42.43**	**0.209**	**45.73**	**41.53**	**0.443**
MCV	**K/μL**	**50.33**	**49.80**	**0.498**	**50.42**	**50.55**	**0.867**
MCH	**%**	**14.78**	**15.15**	**0.276**	**14.60**	**15.18**	**0.041***
MCHC	**K/μL**	**29.43**	**30.53**	**0.310**	**28.93**	**30.08**	**0.0629**
RDW	**%**	**17.65**	**17.68**	**0.948**	**17.05**	**16.53**	**0.371**
PLT	**K/μL**	**387.50**	**336.75**	**0.636**	**514.25**	**406.75**	**0.158**
MPV	**fL**	**4.15**	**4.35**	**0.190**	**4.23**	**4.60**	**0.058**

Table S2. CBC of female and male TRAIL-/- mice 2 weeks post whole thorax x-ray irradiation of 20 Gy treated with TLY012 or control. There were no statistical differences between any CBC values seen between treatment groups at 2 weeks post irradiation (n = 8/treatment/gender) (student's t-tests corrected for FDR with Benjamini-Yekutieli method).

	Unit	Female Control	Female TLY012	P-value	Male Control	Male TLY012	P-value
WBC	**K/μL**	**2.89**	**2.88**	**0.979**	**3.81**	**4.13**	**0.646**
NE#	**K/μL**	**0.88**	**0.77**	**0.693**	**1.10**	**1.32**	**0.557**
LY#	**K/μL**	**1.84**	**0.77**	**0.982**	**2.45**	**2.50**	**0.887**
MO#	**K/μL**	**0.15**	**1.83**	**0.616**	**0.21**	**0.26**	**0.329**
EO#	**K/μL**	**0.021**	**0.18**	**0.195**	**0.038**	**0.035**	**0.927**
NE%	**%**	**34.14**	**26.01**	**0.337**	**28.35**	**29.59**	**0.834**
LY%	**%**	**60.07**	**64.91**	**0.556**	**64.70**	**62.36**	**0.686**
MO%	**%**	**4.81**	**6.17**	**0.254**	**5.54**	**6.42**	**0.212**
EO%	**%**	**0.84**	**2.38**	**0.153**	**1.07**	**1.18**	**0.910**
BA%	**%**	**0.14**	**0.53**	**0.148**	**0.35**	**0.46**	**0.741**
RBC	**M/μL**	**8.95**	**9.44**	**0.547**	**9.58**	**9.09**	**0.487**

HB	**g/dL**	**11.81**	**12.71**	**0.384**	**13.33**	**12.75**	**0.618**
HCT	**%**	**46.14**	**48.46**	**0.580**	**49.74**	**46.99**	**0.489**
MCV	**K/μL**	**51.58**	**51.40**	**0.818**	**51.88**	**51.65**	**0.756**
MCH	**%**	**13.29**	**13.48**	**0.558**	**13.86**	**13.98**	**0.735**
MCHC	**K/μL**	**25.78**	**26.23**	**0.500**	**26.74**	**27.09**	**0.304**
RDW	**%**	**17.28**	**17.10**	**0.751**	**17.46**	**17.85**	**0.311**
PLT	**K/μL**	**513.00**	**558.00**	**0.652**	**615.88**	**595.00**	**0.858**
MPV	**fL**	**4.73**	**4.48**	**0.148**	**4.50**	**4.73**	**0.219**

Table S3. Method used for 3D reconstruction of lung volume in CTAn software (Bruker) Operations listed using terminology for CTAn that were utilized to reconstruct the lung volume of each individual mouse.

Plug-in	Parameters	Result
Thresholding	Global 14-255*	Segment body from air in lungs
Despeckle	2D space, sweep, all except the largest object, image	Remove the noise and the bed
Bitwise Operations	ROI = COPY image	Copy image to ROI
Reload	Image	Reload original image
Thresholding	Global 0-14*	Segment out the lungs
Bitwise Operations	Image = Image and ROI	Image now only contains white pixels belonging to both the image and ROI
Despeckle	3D space, sweep, all except the largest object, image	Image only contains lungs in 3D space, removes fat and outside body
Despeckle	3D space, remove black speckles, volume less than 33 voxels, image	Clean up the image and remove excess black pixels inside the lung
3D Analysis	Basic values, save results as text table	Excel sheet with volume of lungs
3D Model	*.ctm, Double-Time cubes	Builds and saves a 3D model file that can be opened in CTVol

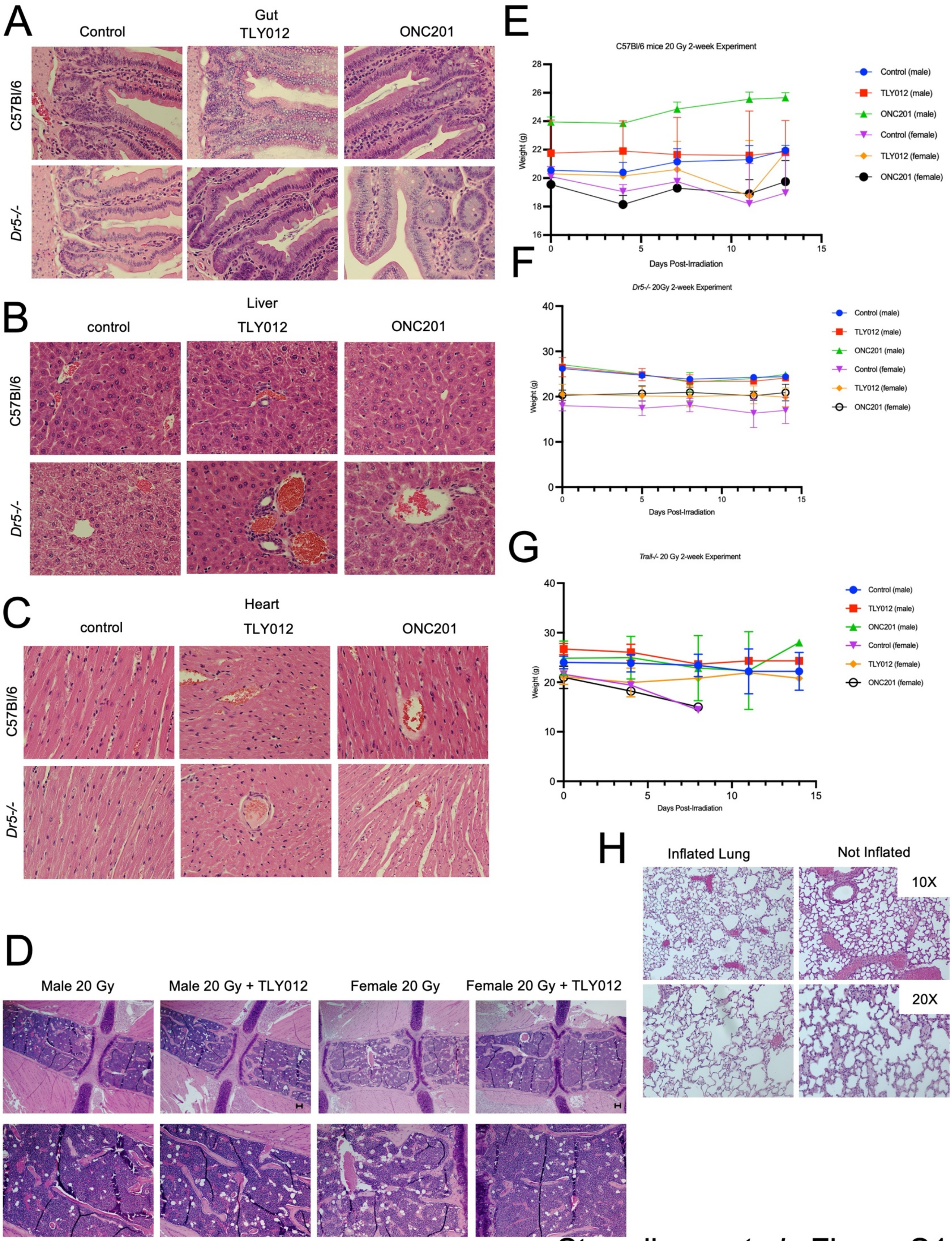

Strandberg *et al.,* Figure S1

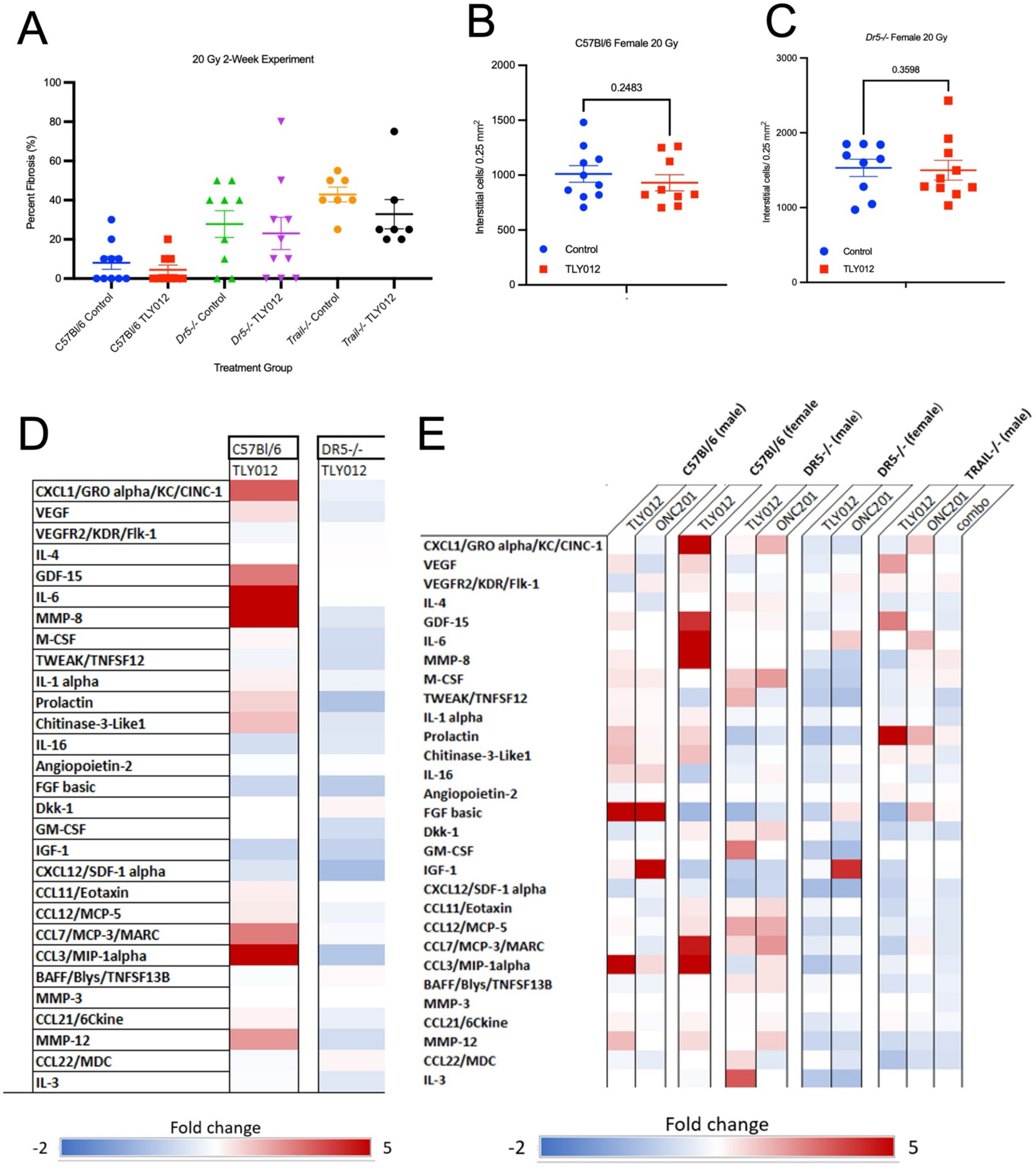

Strandberg *et al.,* Figure S2

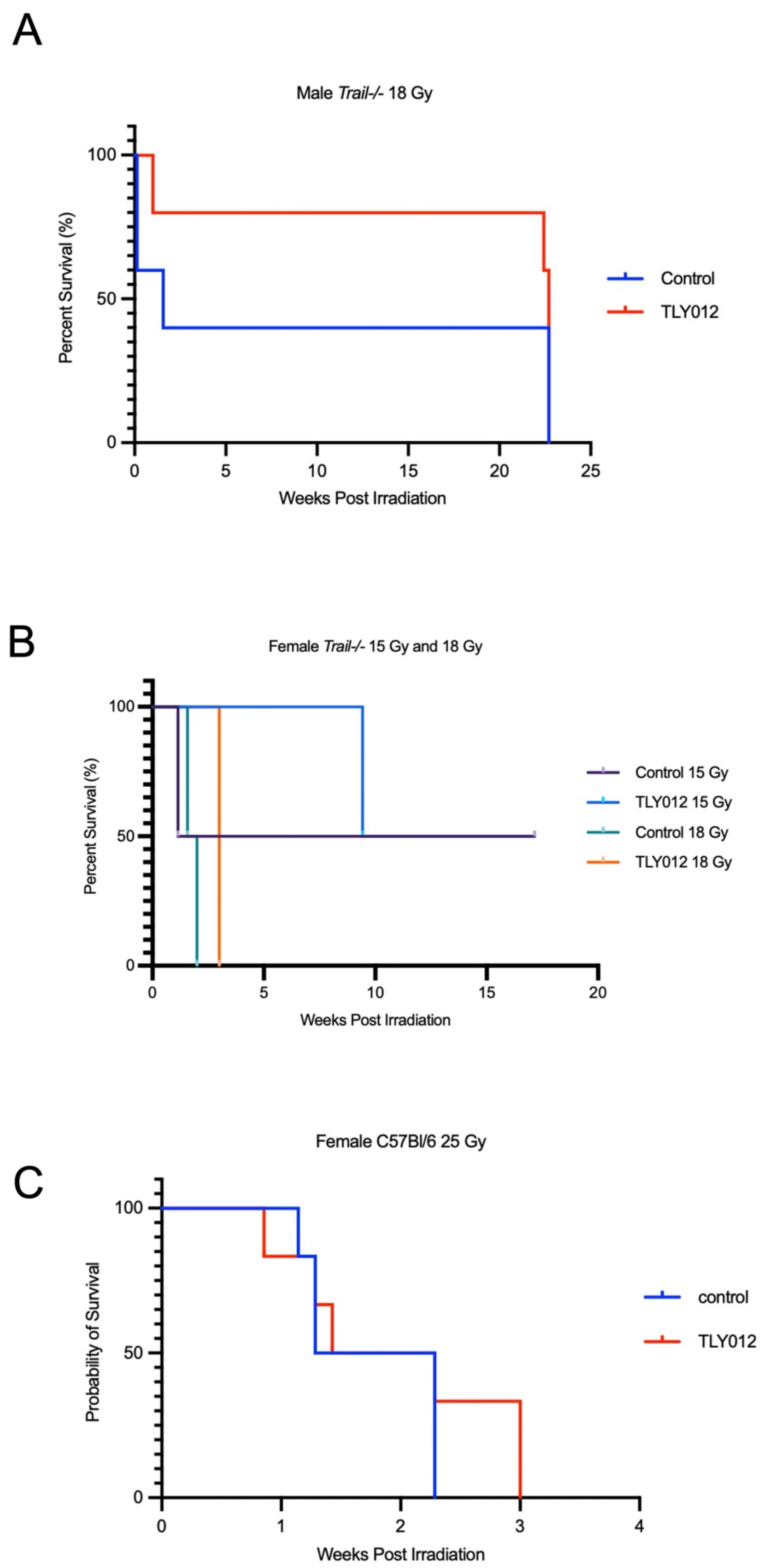

Strandberg *et al.*, Figure S3

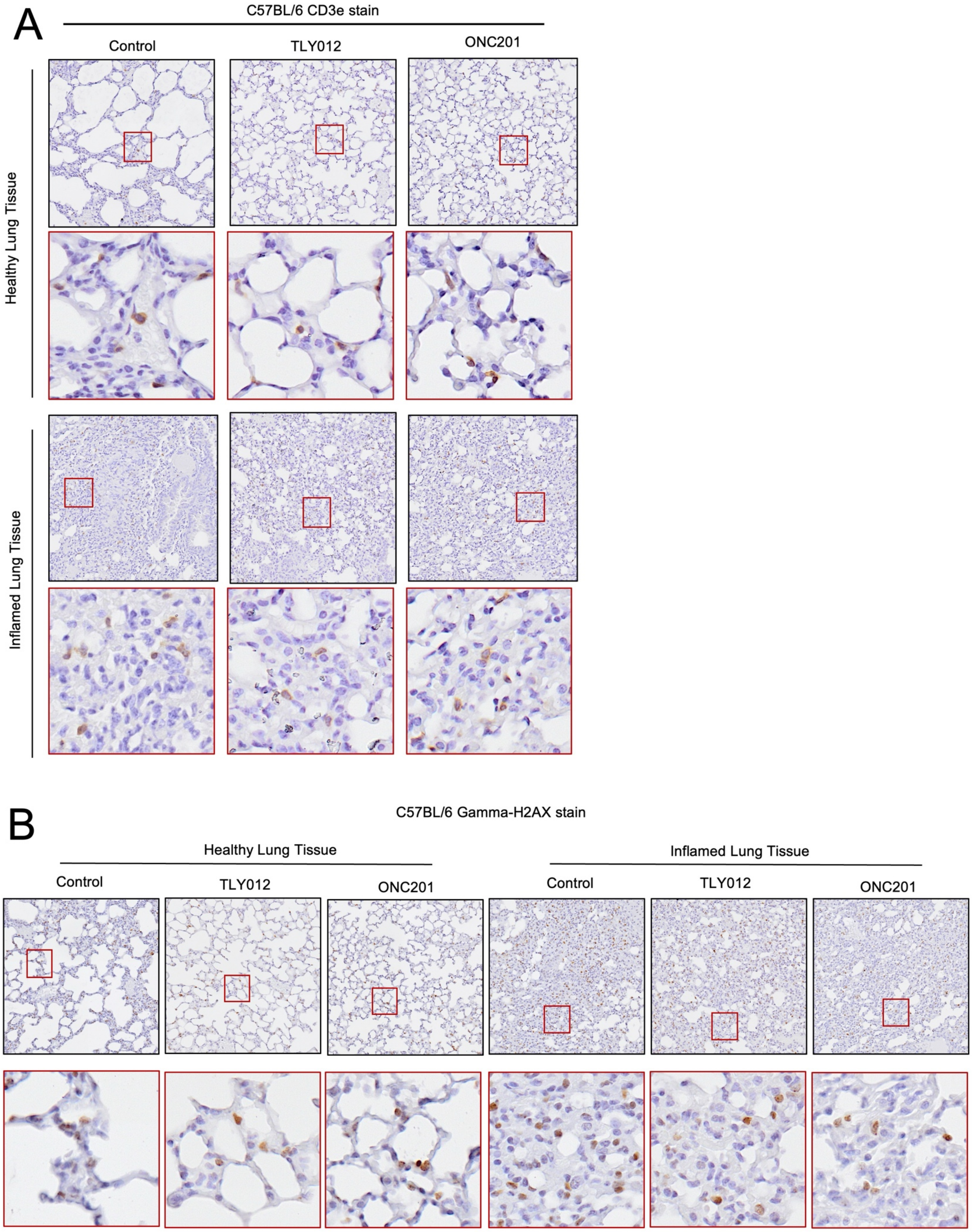

Strandberg *et al.,* Figure S4

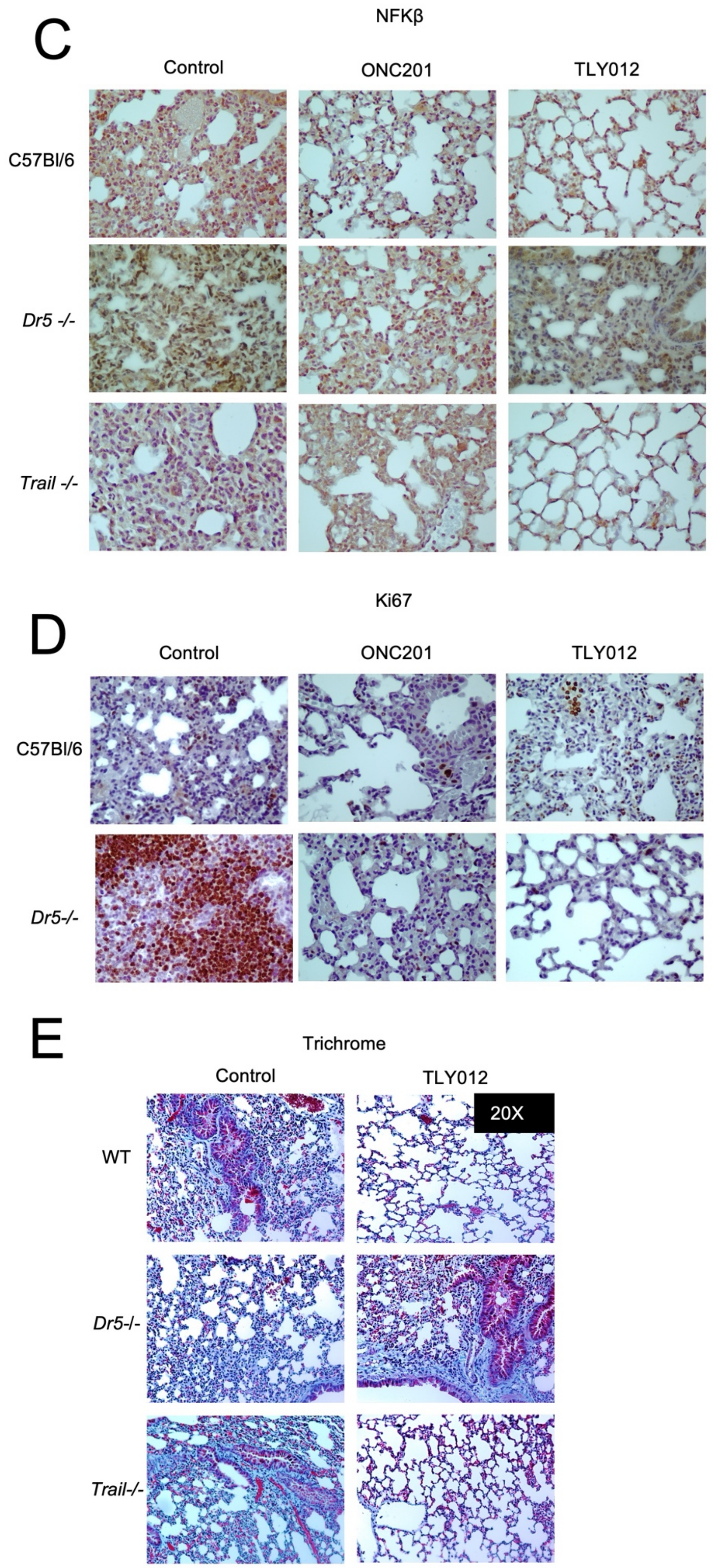

Strandberg *et al.,* Figure S4

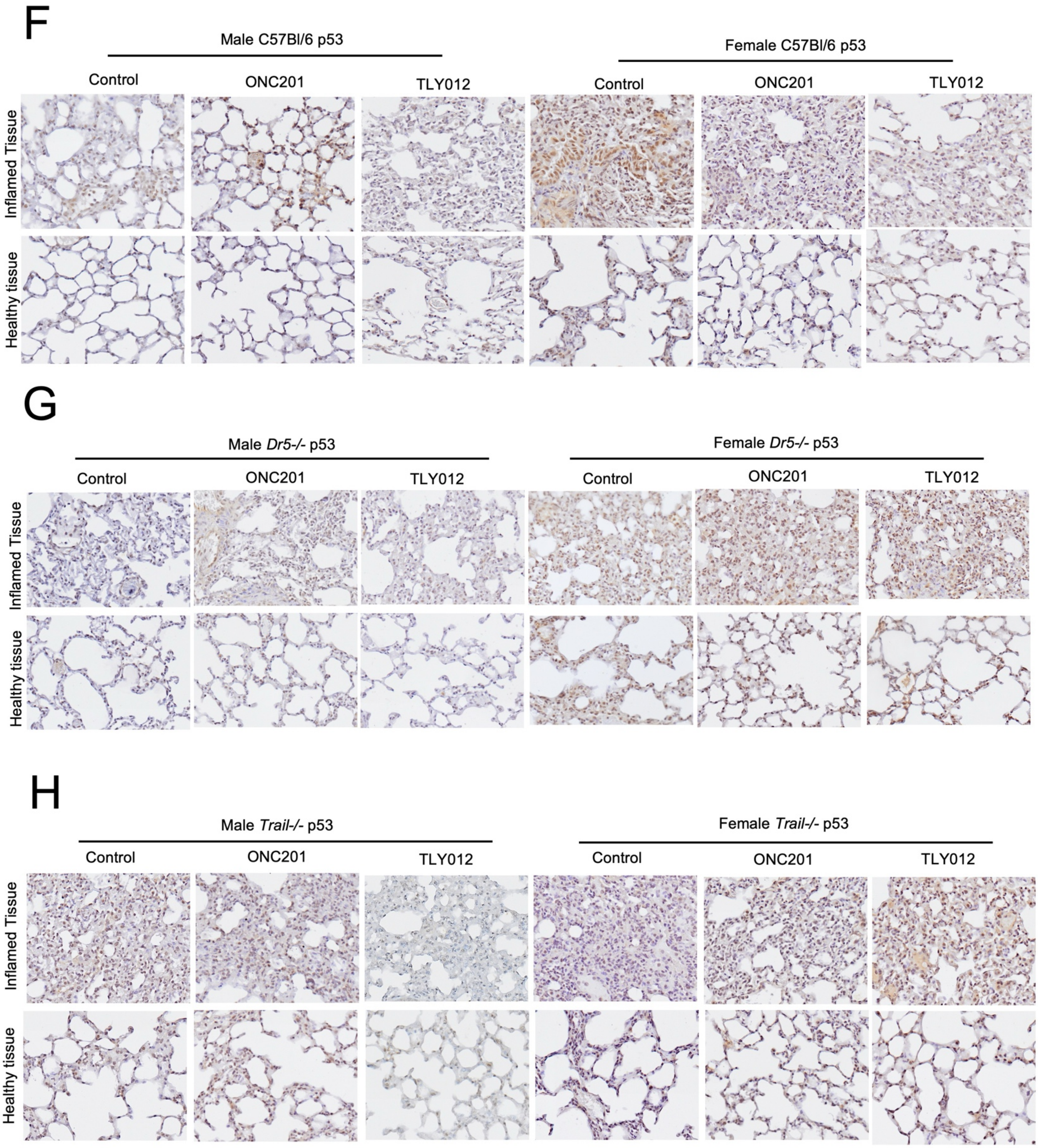

Strandberg *et al.,* Figure S4

I

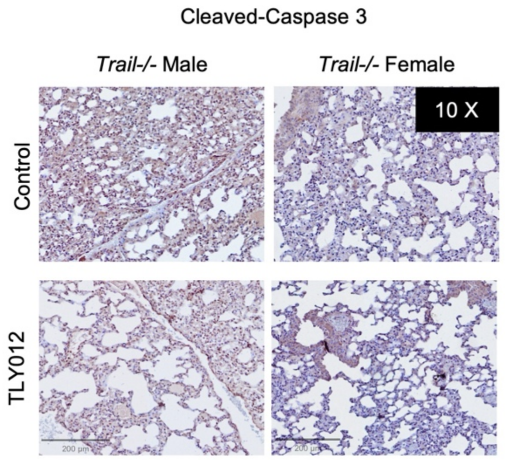

J

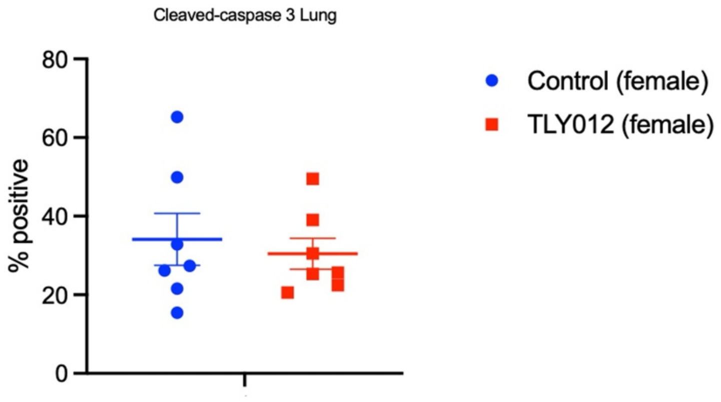

K

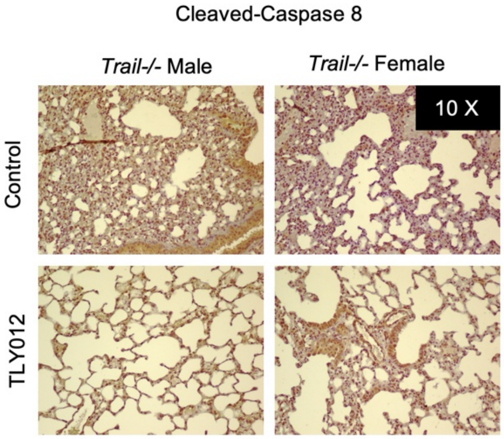

L

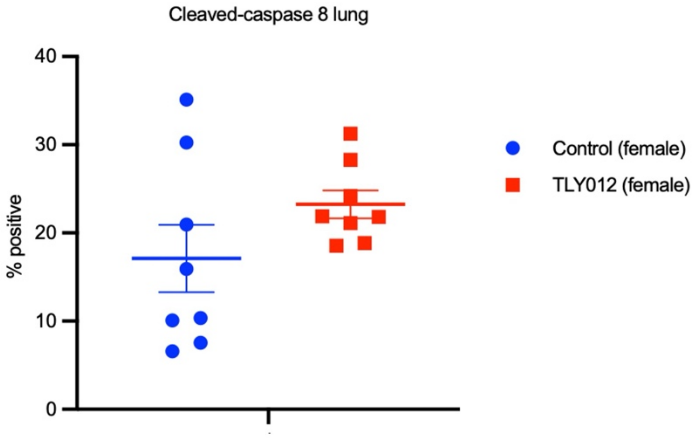

Strandberg *et al.,* Figure S4

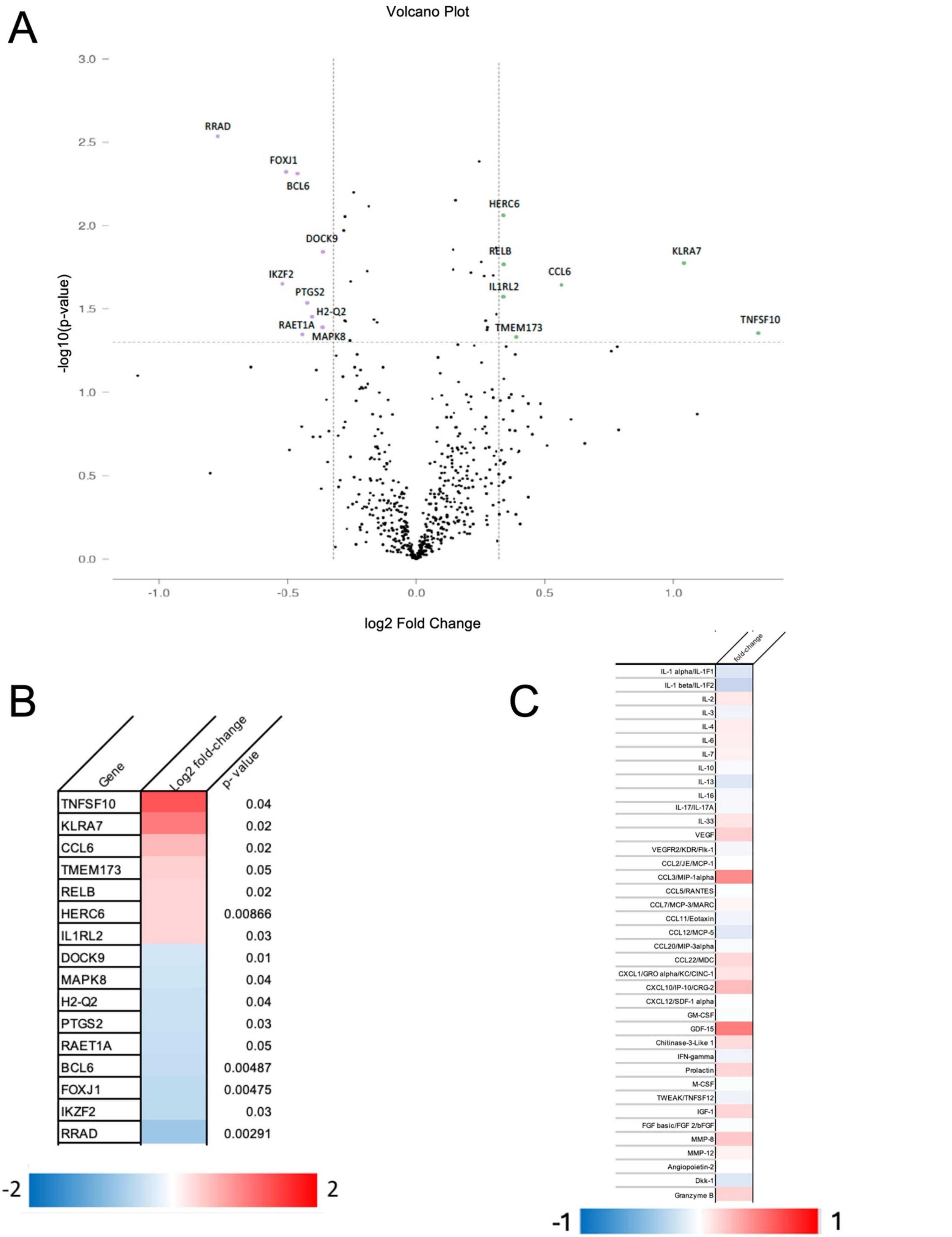

Strandberg *et al.*, Figure S5

D

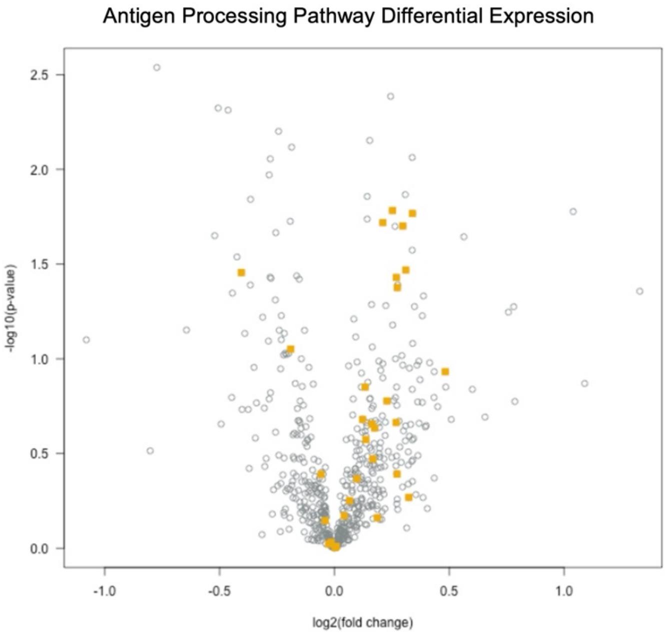

E

Gene	Log2 fold-change	p-value
ICAM1		0.0165
RelB		0.0171
PSMB8		0.0191
H2-Dma		0.0199
H2-Ab1		0.034
H2-Q2		0.0351
H2-Aa		0.0372
CD74		0.0421
NOD1		0.0889
H2-DMb2		0.117
H2-K1		0.141
CCR7		0.167
H2-D1		0.209
FCGR2b		0.217
TAP1		0.221
H2-Eb1		0.232
PSMB9		0.267
H2-DMb1		0.338
FCER1g		0.406
MR1		0.407

-1 1

F

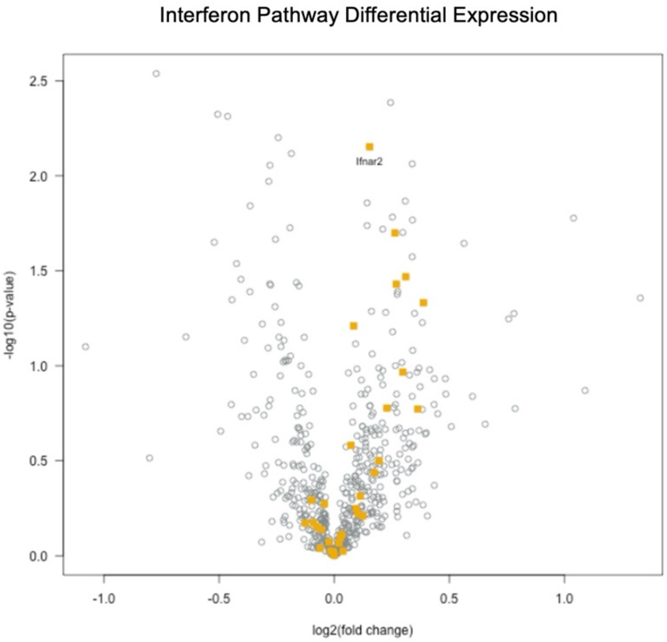

G

Gene	Log2 fold-change	p-value
Ifnar2		0.00704
CXCL16		0.02
H2-Ab1		0.034
H2-Aa		0.0372
TMEM173		0.0466
DDX58		0.0617
IFIT1		0.108
CCR7		0.167
Eomes		0.169
IFNAR1		0.262
IRF8		0.316
IFI44		0.365
IRF7		0.485
IRGM2		0.509
TBK1		0.533
IFIT3		0.565
CIITA		0.6
GBP5		0.617
CD3E		0.669
NLRC5		0.674

-1 1

Strandberg *et al.*, Figure S5

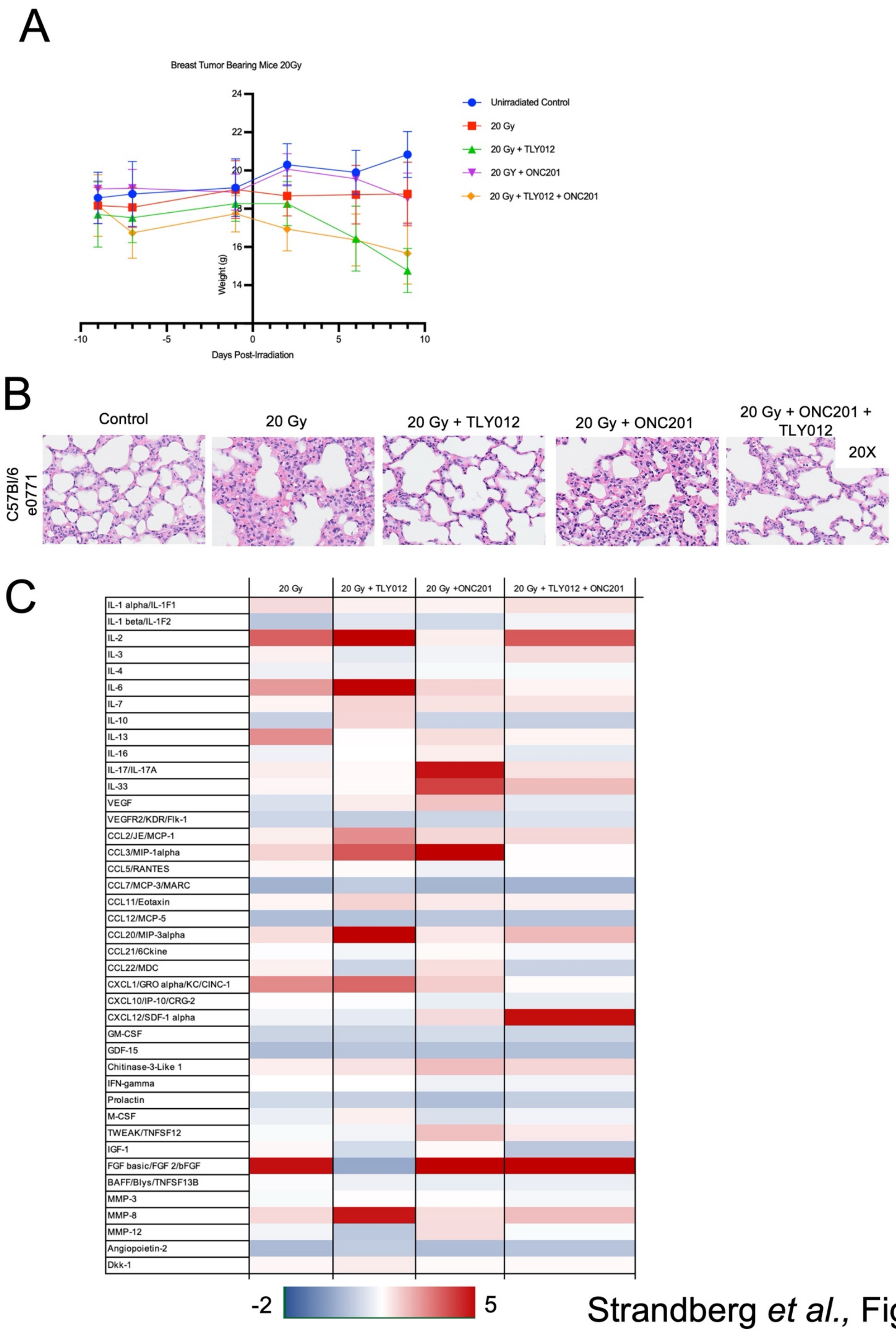

Strandberg *et al.*, Figure S6

A

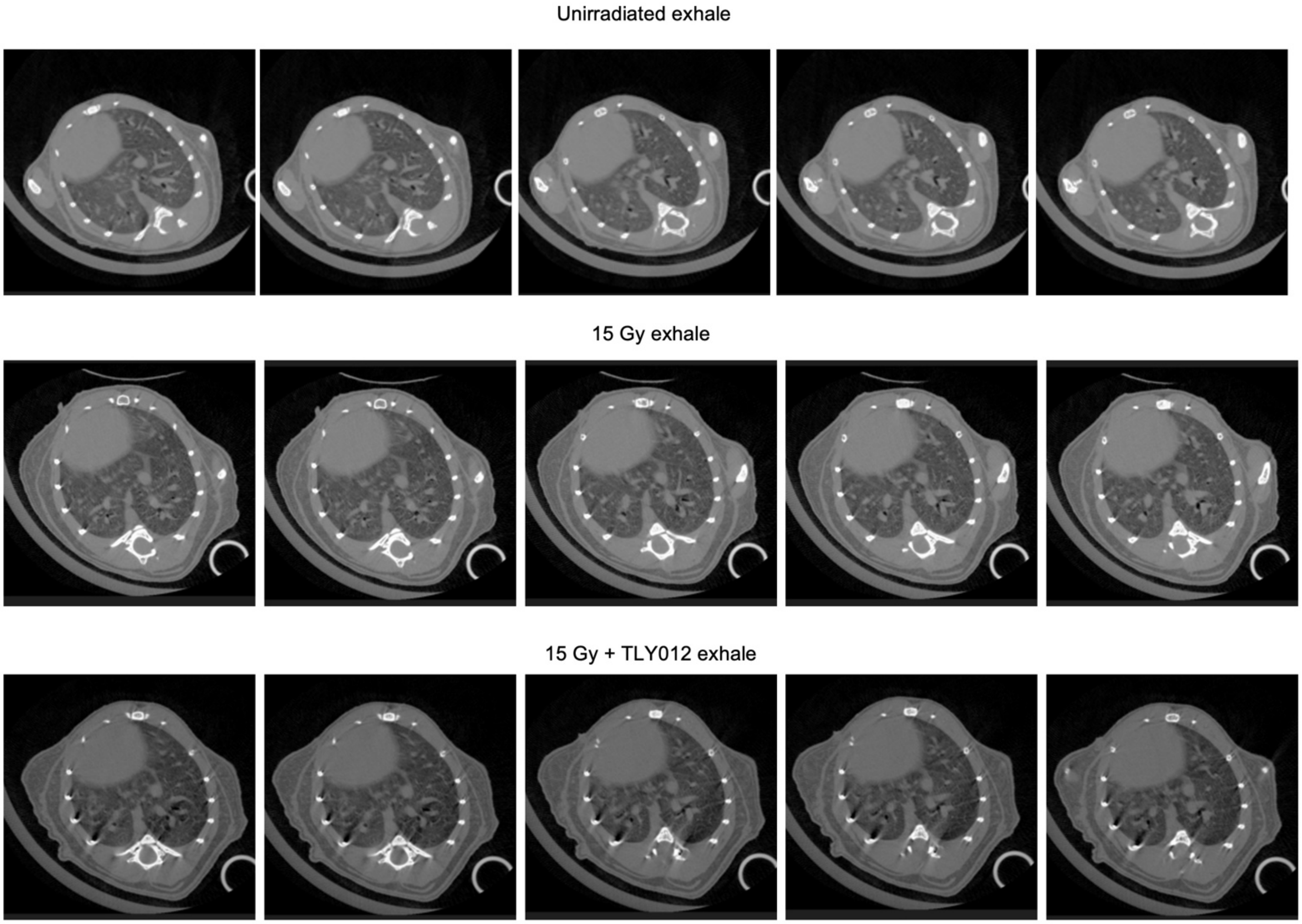

Strandberg *et al.,* Figure S7

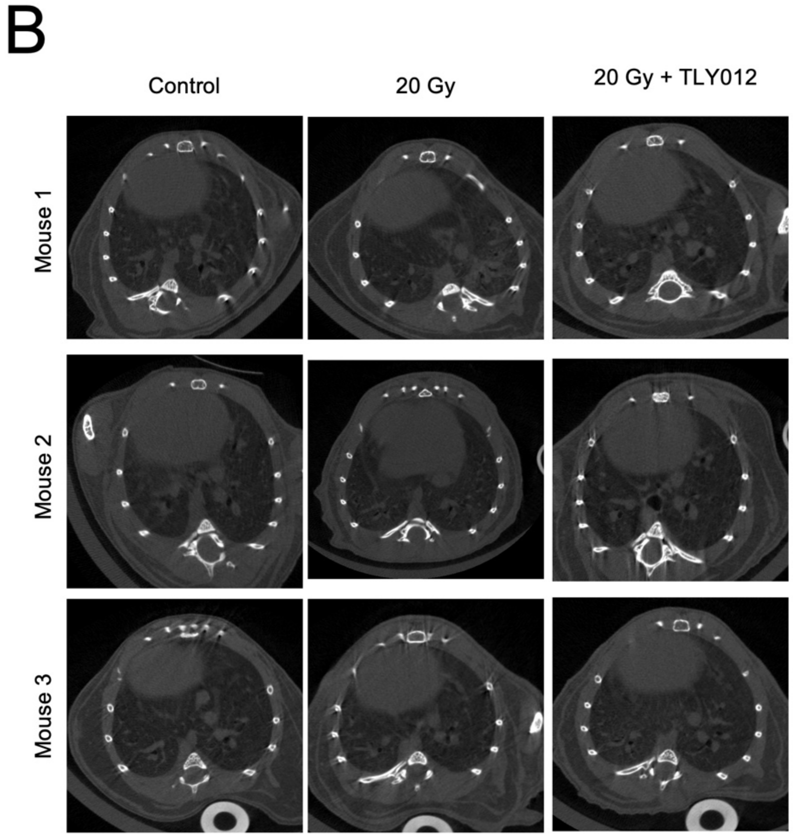

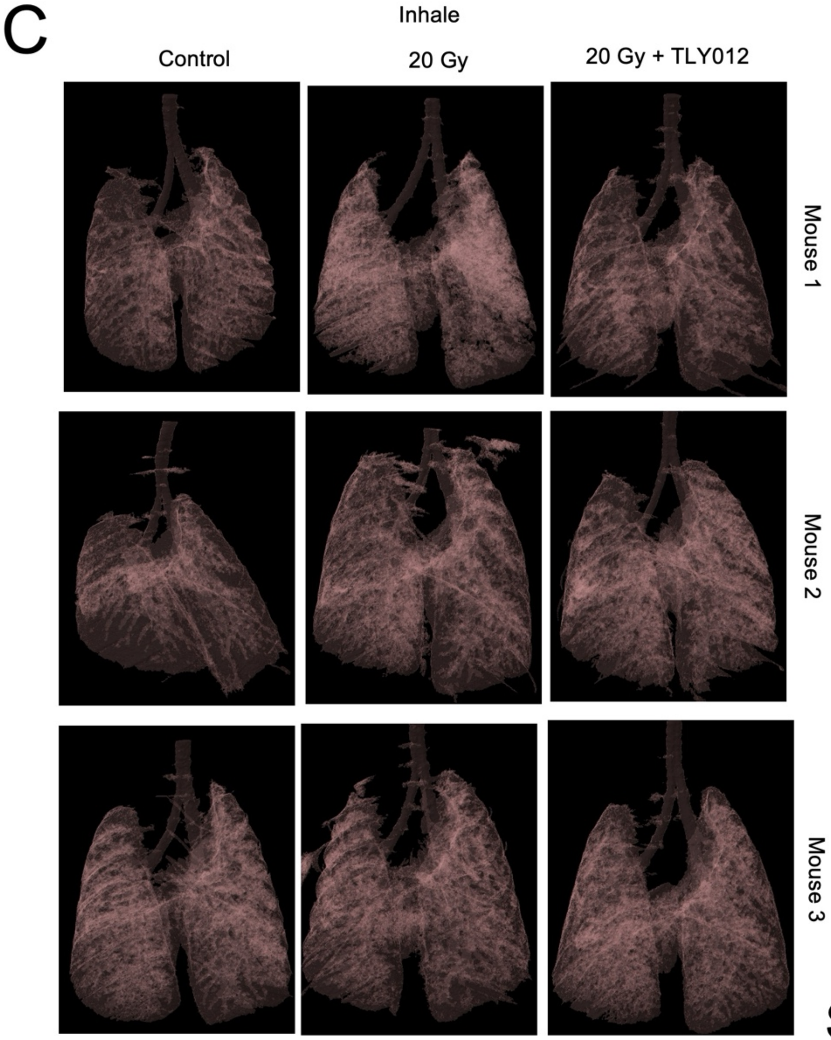

Strandberg *et al.*, Figure S7

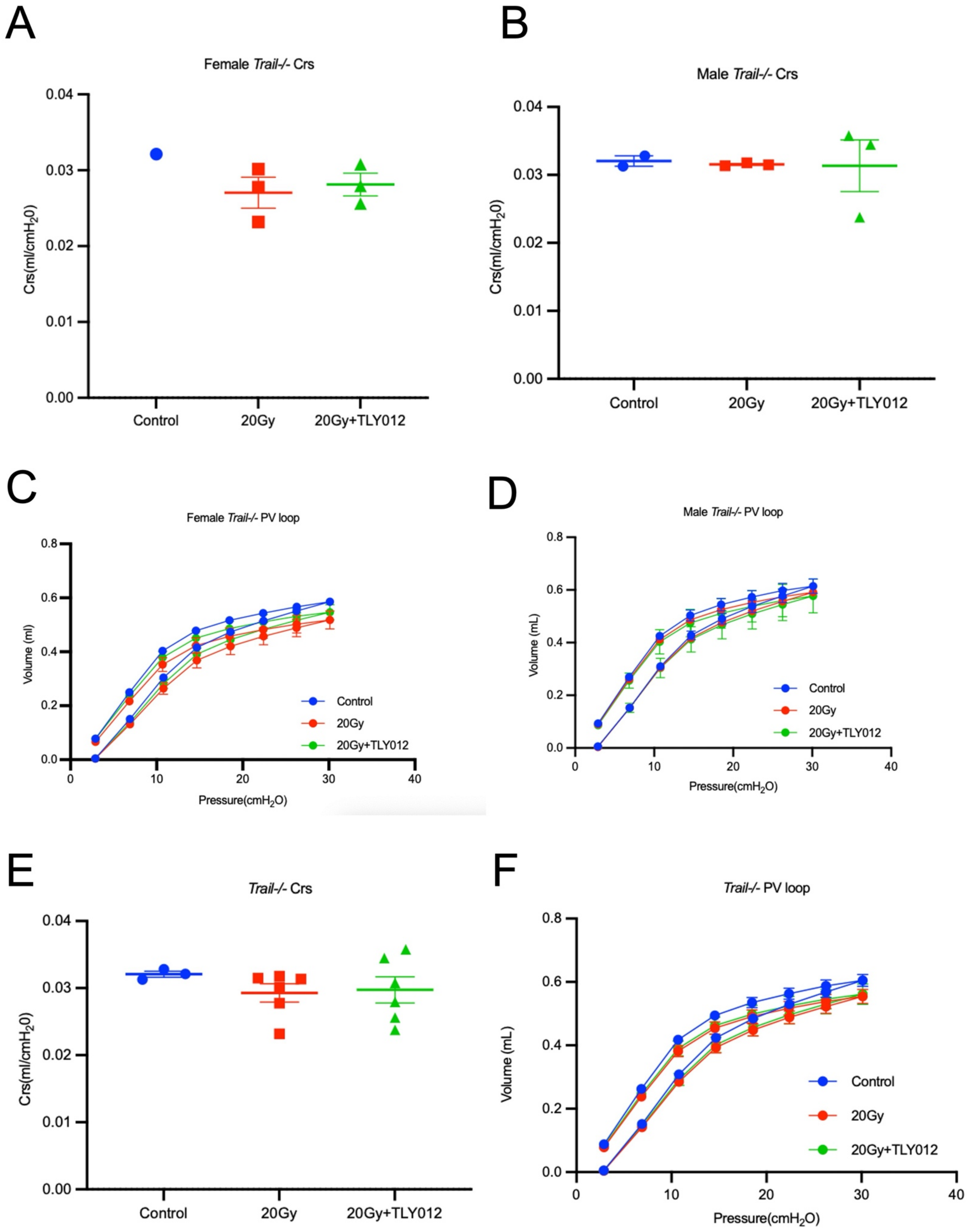

Strandberg *et al.*, Figure S8

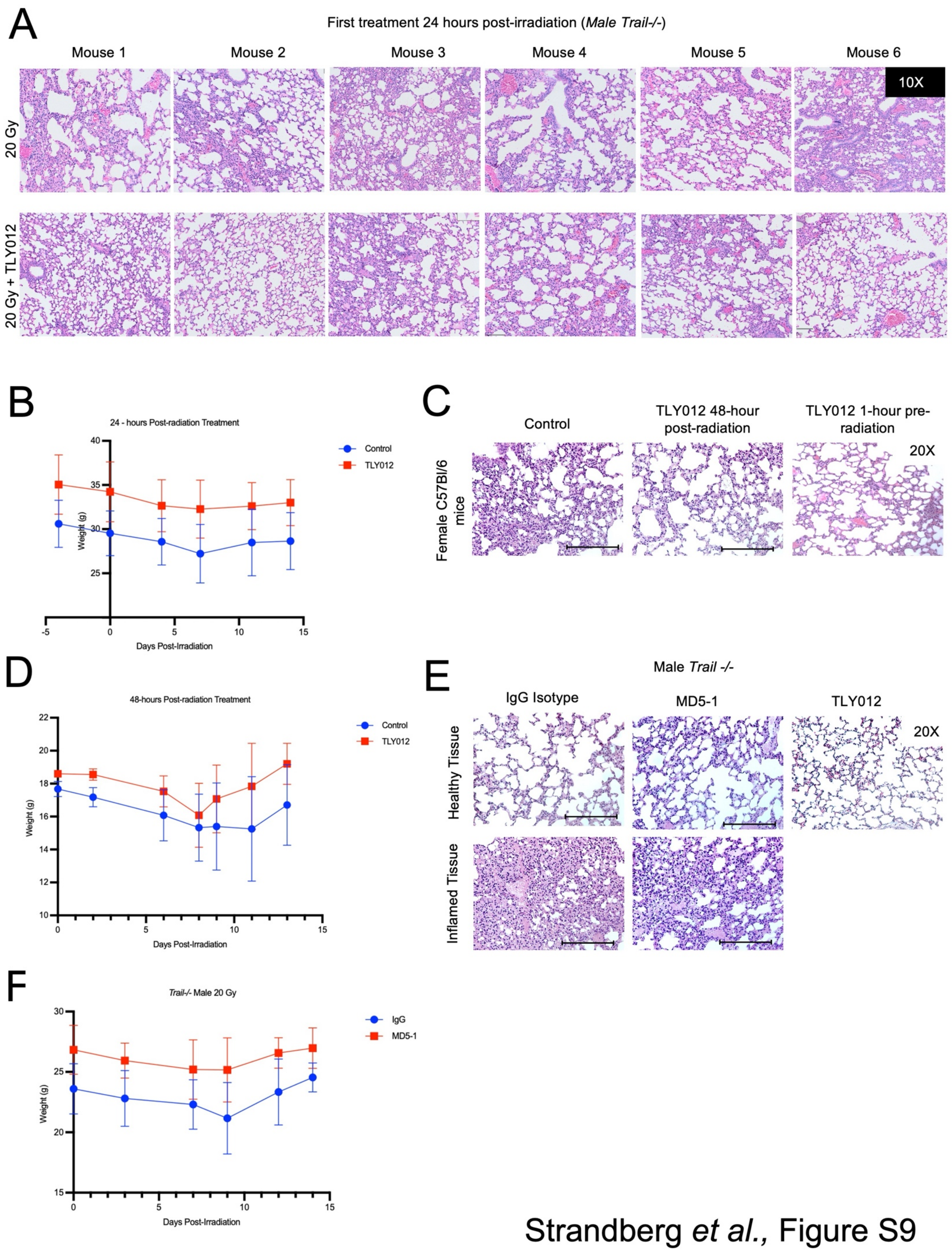

Strandberg *et al.,* Figure S9
